# Risk assessment for colorectal cancer via polygenic risk score and lifestyle exposure: a large-scale association study of East Asian and European populations

**DOI:** 10.1186/s13073-023-01156-9

**Published:** 2023-01-24

**Authors:** Junyi Xin, Mulong Du, Dongying Gu, Kewei Jiang, Mengyun Wang, Mingjuan Jin, Yeting Hu, Shuai Ben, Silu Chen, Wei Shao, Shuwei Li, Haiyan Chu, Linjun Zhu, Chen Li, Kun Chen, Kefeng Ding, Zhengdong Zhang, Hongbing Shen, Meilin Wang

**Affiliations:** 1 Department of Environmental Genomics, Jiangsu Key Laboratory of Cancer Biomarkers, Prevention and Treatment, Collaborative Innovation Center for Cancer Personalized Medicine, Nanjing Medical University, School of Public Health, Nanjing Medical University, 101 Longmian Avenue, Jiangning District, Nanjing, 211166 China; 2 Department of Genetic Toxicology, The Key Laboratory of Modern Toxicology of Ministry of Education, Center for Global Health, School of Public Health, Nanjing Medical University, Nanjing, China; 3 Department of Biostatistics, Center for Global Health, School of Public Health, Nanjing Medical University, Nanjing, China; 4 Department of Oncology, Nanjing First Hospital, Nanjing Medical University, Nanjing, China; 5 Department of Gastroenterological Surgery, Laboratory of Surgical Oncology, Beijing Key Laboratory of Colorectal Cancer Diagnosis and Treatment Research, Peking University People’s Hospital, No. 11 Xizhimen South Street, Xicheng District, Beijing, China; 6 Cancer Institute, Fudan University Shanghai Cancer Center, Shanghai, China; 7 Department of Oncology, Shanghai Medical College, Fudan University, Shanghai, China; 8 Department of Epidemiology and Biostatistics at School of Public Health, Zhejiang University School of Medicine, Hangzhou, China; 9 Cancer Institute, The Second Affiliated Hospital, Zhejiang University School of Medicine, Hangzhou, China; 10 Department of Colorectal Surgery and Oncology, Key Laboratory of Cancer Prevention and Intervention, Ministry of Education, The Second Affiliated Hospital, Zhejiang University School of Medicine, Hangzhou, Zhejiang, China; 11 Cancer Center, Zhejiang University, Hangzhou, Zhejiang, China; 12 Department of Oncology, The First Affiliated Hospital of Nanjing Medical University, Nanjing, China; 13 Department of Epidemiology, Center for Global Health, School of Public Health, Nanjing Medical University, Nanjing, China; 14 The Affiliated Suzhou Hospital of Nanjing Medical University, Suzhou Municipal Hospital, Gusu School, Nanjing Medical University, Suzhou, China

**Keywords:** Colorectal cancer, East Asian, European, Polygenic risk score, Lifestyle

## Abstract

**Background:**

The genetic architectures of colorectal cancer are distinct across different populations. To date, the majority of polygenic risk scores (PRSs) are derived from European (EUR) populations, which limits their accurate extrapolation to other populations. Here, we aimed to generate a PRS by incorporating East Asian (EAS) and EUR ancestry groups and validate its utility for colorectal cancer risk assessment among different populations.

**Methods:**

A large-scale colorectal cancer genome-wide association study (GWAS), harboring 35,145 cases and 288,934 controls from EAS and EUR populations, was used for the EAS-EUR GWAS meta-analysis and the construction of candidate EAS-EUR PRSs via different approaches. The performance of each PRS was then validated in external GWAS datasets of EAS (727 cases and 1452 controls) and EUR (1289 cases and 1284 controls) ancestries, respectively. The optimal PRS was further tested using the UK Biobank longitudinal cohort of 355,543 individuals and ultimately applied to stratify individual risk attached by healthy lifestyle.

**Results:**

In the meta-analysis across EAS and EUR populations, we identified 48 independent variants beyond genome-wide significance (*P* < 5 × 10^−8^) at previously reported loci. Among 26 candidate EAS-EUR PRSs, the PRS-CSx approach-derived PRS (defined as PRS_CSx_) that harbored genome-wide variants achieved the optimal discriminatory ability in both validation datasets, as well as better performance in the EAS population compared to the PRS derived from known variants. Using the UK Biobank cohort, we further validated a significant dose-response effect of PRS_CSx_ on incident colorectal cancer, in which the risk was 2.11- and 3.88-fold higher in individuals with intermediate and high PRS_CSx_ than in the low score subgroup (*P*_trend_ = 8.15 × 10^−53^). Notably, the detrimental effect of being at a high genetic risk could be largely attenuated by adherence to a favorable lifestyle, with a 0.53% reduction in 5-year absolute risk.

**Conclusions:**

In summary, we systemically constructed an EAS-EUR PRS to effectively stratify colorectal cancer risk, which highlighted its clinical implication among diverse ancestries. Importantly, these findings also supported that a healthy lifestyle could reduce the genetic impact on incident colorectal cancer.

**Supplementary Information:**

The online version contains supplementary material available at 10.1186/s13073-023-01156-9.

## Background

Colorectal cancer is one of the most commonly diagnosed cancers and the second leading cause of cancer death worldwide, with over 1.8 million new cases and 0.9 million deaths in 2020 [1]. Cumulative evidence has demonstrated that colorectal cancer is caused by environmental factors (e.g., lifestyle), genetic factors, and their interactions [2]. Although environmental risk factors contribute the most, genetic variants can separately explain approximately 7–16% of heritability for colorectal cancer among European (EUR) and East Asian (EAS) populations, indicating the vital role of variants in the development of colorectal cancer [3, 4].

In the past decades, genome-wide association studies (GWASs) have identified over 100 single nucleotide polymorphisms (SNPs) associated with the risk of colorectal cancer [5–7]. Although each of these risk variants contributes a small effect on colorectal cancer risk, the polygenic risk score (PRS), a method that combines the weak effect of these known or genome-wide variants, has been found to be an efficient tool for identifying individuals at high risk of developing colorectal cancer risk [8–10]. However, most PRSs were developed and optimized based on the GWAS data of EUR ancestry and had a limited discriminating ability among other populations (e.g., EAS) [10, 11]. Therefore, it is urgent to construct a trans-ancestry PRS that can improve the ability of colorectal cancer risk prediction in diverse populations.

Unhealthy lifestyles have been known to be associated with an increased risk of colorectal cancer, while healthy lifestyle habits show inverse associations [12]. In particular, accumulating evidence indicated that among individuals with high genetic risk, cancer risk can be attenuated by adherence to a healthy lifestyle, such as colorectal cancer [13], as well as our previous studies in gastric cancer [14] and lung cancer [15].

**Fig. 1:**
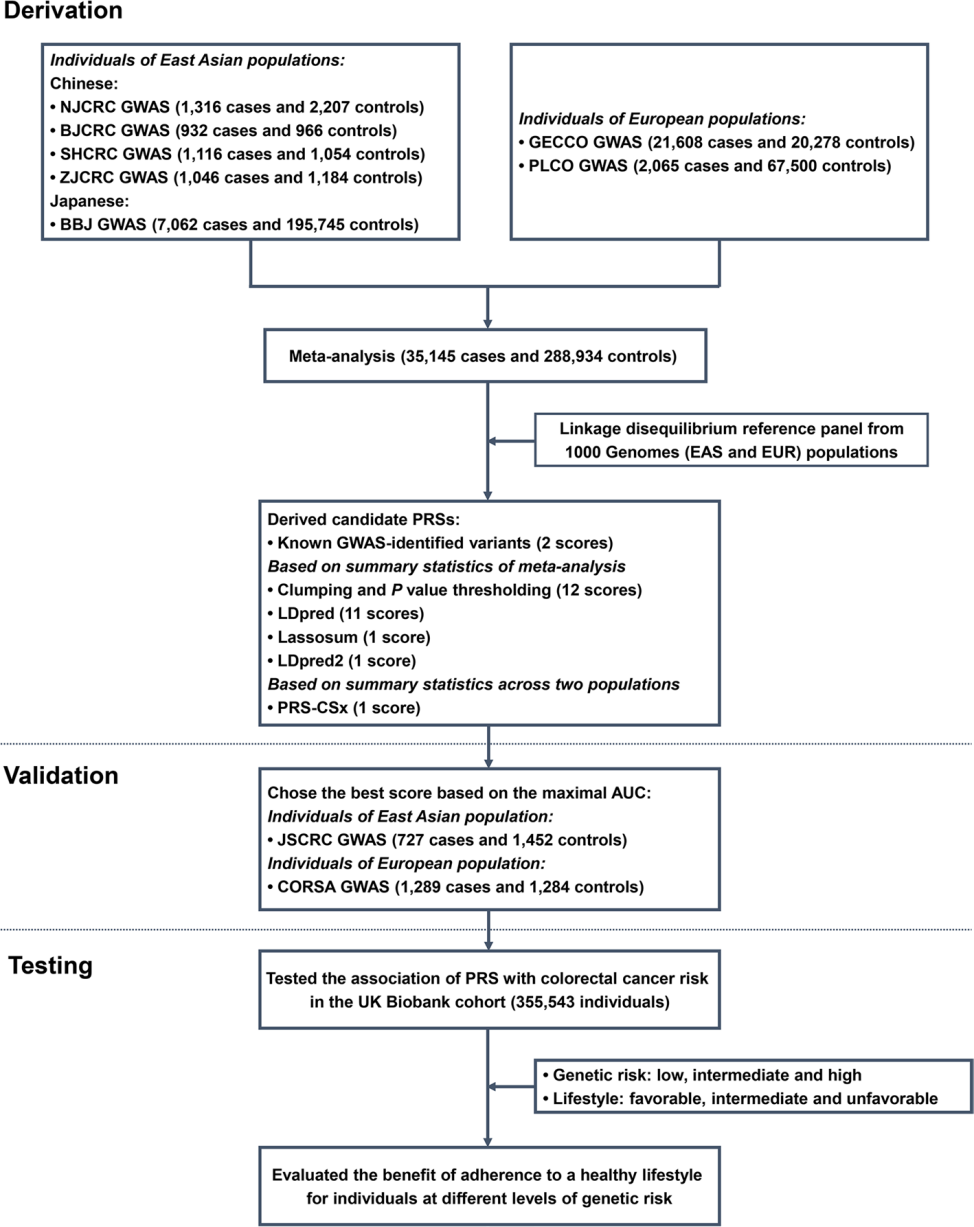
Summary of the study design. GWAS, genome-wide association study; EAS, East Asian population; EUR, European population; PRS, polygenic risk score; AUC, area under the receiver operating characteristics curve; PLCO, Prostate, Lung, Colorectal and Ovarian cancer screening trial; GECCO, Genetics and Epidemiology of Colorectal Cancer Consortium; CORSA, Colorectal Cancer Study of Austria; BBJ, BioBank Japan Project

In this study, we performed a large-scale meta-analysis of EAS and EUR populations, to identify common genetic variants associated with colorectal cancer risk across the two ethnic groups. Subsequently, we aimed to develop a novel EAS-EUR PRS that can be used to stratify colorectal cancer risk in diverse populations, and further evaluate the benefit of adherence to a healthy lifestyle stratified by different levels of genetic risk for developing colorectal cancer in a longitudinal cohort (Fig. 1).

## Methods

### Study participants

#### Case-control studies of derivation stage

##### EAS of the Chinese population

The subjects of four independent Chinese colorectal cancer GWAS (Additional file 1: Table S1 and Fig. S1) were recruited from the National ColoRectal Cancer Cohort (NCRCC), including NJCRC GWAS [1316 cases and 2207 controls [16], being part of the Genetics and Epidemiology of Colorectal Cancer Consortium (GECCO)], BJCRC GWAS (932 cases and 966 controls) [17], SHCRC GWAS (1116 cases and 1054 controls), and ZJCRC GWAS (1046 cases and 1184 controls). The detailed information is described in Additional file 1: Supplementary Materials.

##### EAS of the Japanese population

All participants of the Japanese GWAS were collected in the BioBank Japan Project (BBJ), and the population details have been published in a previous study [18]. We obtained the GWAS summary statistics of colorectal cancer (7062 cases and 195,745 controls) from the JENGER website.

##### EUR population (GECCO)

The GWAS datasets of GECCO consortia were deposited in the database of Genotypes and Phenotypes (dbGaP, phs001315.v1.p1; phs001415.v1.p1 and phs001078.v1.p1). All cases were confirmed by medical records, pathologic reports, cancer registries, or death certificates. The population details have been published in previous studies [5, 6]. After individual-level quality control (Additional file 1: Supplementary Materials), a total of 21,608 cases and 20,278 controls, which did not include datasets of Prostate, Lung, Colorectal, and Ovarian (PLCO) and Colorectal Cancer Study of Austria (CORSA), were retained for analysis.

##### EUR population (PLCO)

The PLCO cancer screening trial is a cohort study that aims to evaluate the accuracy and reliability of screening methods for prostate, lung, colorectal, and ovarian cancer [19], and the detailed information was described in our previous study [20]. We obtained the up-to-date GWAS summary statistics of colorectal cancer (2065 cases and 67,500 controls; October 18, 2022) in the EUR population from the PLCOjs website [21]. This study was approved by the ethics committees of the PLCO consortium providers (#PLCO-84).

#### Case-control studies of the validation stage

##### EAS of the Chinese population

The confirmed cases from the JSCRC study were consecutively recruited from hospitals in Jiangsu province, China. The cancer-free control subjects were selected from individuals receiving routine physical examination at hospitals or those participating in community screening for non-communicable diseases in Jiangsu province. A total of 727 cases and 1452 controls were finally included in this study.

##### EUR population (CORSA)

The CORSA dataset included colorectal cancer and adenoma cases and colonoscopy-negative controls. Controls received a complete colonoscopy and were free of colorectal cancer or polyps [22]. We accessed the CORSA genotype data from dbGaP (phs001415.v1.p1) and kept 1289 cases and 1284 controls for subsequent analysis after the individual-level quality control process (Additional file 1: Supplementary Materials).

#### Longitudinal cohort of the testing stage

The UK Biobank cohort is a prospective, population-based study, which recruited 502,528 adults aged 40–69 years from the general population between April 2006 and December 2010 [23]. After individual-level quality control (Additional file 1: Supplementary Materials), a total of 355,543 participants were retained for our analysis (Additional file 1: Table S2) [24]. The follow-up time was calculated from baseline assessment to the first diagnosis of colorectal cancer [International Classification of Diseases, 10th revision (ICD-10) codes with C18-C20], loss to follow-up, and death or last follow-up (December 14, 2016). This study was conducted using the UK Biobank Resource under Application #45611.

##### GWAS meta-analysis of colorectal cancer 

The genotyping, imputation, and SNP-level quality control procedures of all GWAS datasets are described in Additional file 1: Supplementary Materials. We used a multivariable logistic regression model to estimate the odds ratios (ORs) and 95% confidence intervals (CIs) for each SNP with the adjustment of sex, age, and principal components of ancestry, separately for each individual-level GWAS dataset.

We then performed a meta-analysis based on the summary statistics derived from EAS and EUR populations of derivation datasets (35,145 cases and 288,934 controls in total) using the inverse variance-weighted fixed-effects model, implemented by the METAL software [25]. After obtaining the summary statistics of the meta-analysis, we excluded SNPs if they (i) had substantial heterogeneity identified among studies (*P* value for heterogeneity test < 0.001) and (ii) did not pass filters in both EAS and EUR populations, a total of 4.7 million SNPs were retained for further analysis, and variants at *P* value < 5 × 10^−8^ were considered to be genome-wide significant. In the previously reported regions, genome-wide significant SNPs with *P*_conditional_ < 5 × 10^−8^ were considered as novel variants using conditional analysis with the Genome-wide Complex Trait Analysis (GCTA) software conditioning on the known SNPs [26].

##### Calculation of PRS

We calculated PRS to aggregate the weak effect of individual SNP [8], based on the following formula: $\textrm{PRS}=\sum_{i=1}^n{\beta}_i{\textrm{SNP}}_{\textrm{i}}$, where *n* means the number of SNPs, SNP_*i*_ and *β*_*i*_ are the number of risk alleles (i.e., 0, 1, 2), and weight carried by the *i*th SNP. The EAS-ancestry (Additional file 1: Table S3) and EUR-ancestry PRSs [10] were constructed using GWAS-reported variants. Furthermore, the development of candidate EAS-EUR PRSs was determined by five different approaches (Additional file 1: Supplementary Materials), including clumping and *P* value thresholding (i.e., C+T) approach (12 scores) [27], LDpred (11 scores) [28], lassosum (1 score) [29], LDpred2 (1 score) [30], and PRS-CSx methods (1 score) [31]. The 1000 Genomes EAS and EUR populations (Phase 3; 769 individuals) were used as a reference panel. The proportions of the different ethnic groups in the reference panel were consistent with those in the meta-analysis of EAS and EUR GWASs.

##### Calculation of lifestyle score

We calculated healthy lifestyle scores based on the eight lifestyle factors [32], including body mass index (BMI), tobacco smoking, alcohol consumption, waist-to-hip ratio (WHR), physical activity, sedentary time, red and processed meat intake, and vegetable and fruit intake (Additional file 1: Table S4). Each lifestyle factor was given a score of 0 or 1, with 1 representing the healthy behavior category, and the sum of the eight scores was used as the healthy lifestyle score. The detailed information is described in Additional file 1: Supplementary Materials.

##### Estimation of 5-year absolute risk

We estimated individual 5-year absolute risk for developing colorectal cancer by combining the relative risk (incorporating genetic risk and lifestyle) with the incidence rate of colorectal cancer and the mortality rate for all causes except for colorectal cancer [9], and the exact details of the calculations were described in our previous study [16].

##### Statistical analysis

The population structure was estimated using the EIGENSOFT software [33], and the Manhattan plot and quantile-quantile plot based on the -log_10_ (*P* value) were created by using the R package *qqman* (https://cran.r-project.org/web/packages/qqman/index.html). We evaluated the discriminatory ability of PRSs derived from different approaches described above using the crude and covariates-adjusted area under the receiver operating characteristics curve (AUC) via the R package *RISCA* [34].

In the UK Biobank cohort, the Cox proportional hazards model was used to estimate the hazard ratios (HRs) and 95% CIs after adjusting for corresponding confounding factors. We compared the difference in the distribution of PRS between two or more groups by the Wilcoxon or Kruskal-Wallis tests. Participants were classified into ten equal subgroups according to the decile distribution of PRS and categorized into low (bottom 10%), intermediate (10–90%), and high genetic risk (top 10%) subgroups for group comparisons. Similarly, participants were classified into unfavorable (0 and 1 score), intermediate (2 and 3 score), and favorable (≥ 4 score) lifestyle subgroups based on lifestyle scores ranging from 0 to 8. The log-rank test was used to evaluate the difference in cumulative incidence (one minus the Kaplan-Meier estimate) stratified by different levels of PRS or lifestyle scores. The incidence proportion and 95% CI in each group were estimated by the exact Poisson test. The R package *Shiny* (https://cran.r-project.org/web/packages/shiny/) was used to construct the colorectal cancer risk prediction web server, which was freely available and open source.

In addition, to assess the robustness of the results, we performed the following sensitivity analyses: (i) excluded incident colorectal cancer cases that had occurred during the first year of follow-up; (ii) evaluated the associations using ancestry-corrected PRS: briefly, fit a linear regression model using the first ten principal components of ancestry to predict PRS, and the residual from this model was used to create ancestry-corrected PRS; (iii) healthy lifestyle categories were reclassified to unfavorable (0, 1, and 2 score), intermediate (3 and 4 score), and favorable (≥ 5 score) lifestyle groups; and (iv) excluded non-colorectal cancer participants with other cancers that occurred during the time of follow-up.

All other statistical analyses were performed using the R software (version 3.6.1, https://cran.r-project.org/), and a two-sided *P* value less than 0.05 was considered as significant.

## Results

### EAS-EUR GWAS meta-analysis of colorectal cancer

The combined EAS-EUR GWAS dataset of colorectal cancer comprised a total of 35,145 cases and 288,934 controls, and there was no residual population stratification observed via genomic control inflation factors (lambda = 1.002; Additional file 1: Fig. S2).

In total, we identified 48 independent SNPs [linkage disequilibrium (LD) *r*^2^ < 0.1] that were significantly associated with colorectal cancer risk beyond genome-wide significance (*P* < 5 × 10^−8^; Table 1; Additional file 1: Fig. S3). We found that all of these SNPs were located within 1 Mb of well-identified regions reported by previous GWASs, while one novel risk variant (LD *r*^2^ < 0.1 with the previously reported SNPs) was found to be independently associated with colorectal cancer risk in conditional analyses on GWAS-reported risk variants [rs7623129 (3p14.1), OR_conditional_ = 1.06, *P*_conditional_ = 1.18 × 10^−8^; Additional file 1: Table S5]. Especially, functional annotation showed that rs7623129 overlapped with the enhancer histone mark and DNAse hypersensitivity site, indicating that it may be involved in the development of colorectal cancer by regulating the expression of nearby *ADAMTS9* (Additional file 1: Table S6).

**Table 1:** Summary of 48 independent SNPs from the meta-analysis of GWASs in East Asian and European populations

Locus	Chr	SNP	Position^a^	Allele^b^	RAF^c^		OR (95% CI)^d^	*P*^d^	*P*_het_^e^
					EAS	EUR			
1q25.3	1	rs6424881	182986765	C/T	0.482	0.587	1.08 (1.06, 1.10)	3.05E−14	0.530
1q41	1	rs12140604	222159150	G/C	0.204	0.223	1.09 (1.07, 1.12)	3.49E−14	0.145
3p14.1	3	rs7623129	64624426	C/T	0.455	0.534	1.06 (1.04, 1.08)	2.68E−08	0.714
3q13.2	3	rs72942485	112999560	G/A	0.948	0.991	1.16 (1.11, 1.23)	4.47E−09	0.639
3q22.2	3	rs58383609	133735742	C/A	0.592	0.853	1.07 (1.05, 1.10)	1.54E−08	0.677
4q24	4	rs1909122	106127004	T/C	0.453	0.645	1.09 (1.06, 1.12)	2.79E−11	0.097
5p13.1	5	rs72748467	40262840	T/C	0.042	0.245	1.09 (1.06, 1.12)	7.91E−10	0.735
5q31.1	5	rs519705	134462596	G/A	0.369	0.540	1.07 (1.05, 1.10)	8.04E−12	0.001
5q31.1	5	rs7729156	134507139	T/C	0.305	0.447	1.09 (1.06, 1.11)	1.40E−15	0.411
5q32	5	rs2302274	149546426	G/A	0.596	0.459	1.06 (1.04, 1.08)	1.93E−09	0.839
6p21.33	6	rs2071590	31539768	G/A	0.759	0.626	1.06 (1.04, 1.08)	1.68E−08	0.033
7p12.3	7	rs6948177	47510741	G/A	0.895	0.668	1.06 (1.04, 1.09)	3.98E−08	0.932
7p13	7	rs7810512	45150331	A/C	0.744	0.747	1.07 (1.05, 1.09)	9.87E−09	0.301
8q23.3	8	rs2450114	117623719	A/G	0.146	0.115	1.09 (1.06, 1.12)	1.46E−08	0.003
8q23.3	8	rs2015069	117639532	T/C	0.582	0.859	1.09 (1.06, 1.12)	5.65E−12	0.458
8q23.3	8	rs28668628	117679601	C/T	0.013	0.113	1.12 (1.07, 1.16)	2.12E−08	0.629
8q24.21	8	rs79122086	128397907	G/T	0.203	0.123	1.12 (1.08, 1.16)	1.43E−08	0.009
8q24.21	8	rs6470510	128429660	T/C	0.158	0.187	1.11 (1.09, 1.14)	5.02E−17	0.032
9p21.3	9	rs1537372	22103183	G/T	0.482	0.581	1.07 (1.05, 1.10)	1.28E−12	0.104
10p14	10	rs827385	8705799	T/A	0.635	0.632	1.10 (1.08, 1.12)	7.23E−18	0.001
10q22.3	10	rs704017	80819132	G/A	0.279	0.540	1.12 (1.08, 1.15)	3.67E−13	0.597
10q24.2	10	rs17578367	101344167	A/G	0.179	0.198	1.07 (1.05, 1.10)	2.39E−08	0.887
10q25.2	10	rs12241008	114280702	C/T	0.300	0.094	1.14 (1.11, 1.17)	5.36E−21	0.683
10q25.2	10	rs11196170	114722621	A/G	0.682	0.255	1.07 (1.04, 1.09)	3.19E−08	0.013
11q12.2	11	rs174598	61621194	G/A	0.432	0.643	1.06 (1.04, 1.09)	2.27E−08	0.236
11q13.4	11	rs11236148	74264335	G/A	0.923	0.915	1.10 (1.07, 1.14)	3.52E−08	0.243
11q13.4	11	rs4944913	74321349	A/G	0.680	0.662	1.08 (1.06, 1.11)	2.17E−13	0.885
11q13.4	11	rs6592590	74381029	T/C	0.352	0.406	1.06 (1.04, 1.08)	4.57E−08	0.180
12p13.32	12	rs3217840	4394877	T/C	0.963	0.604	1.08 (1.05, 1.10)	1.50E−09	0.108
12q12	12	rs908664	43133634	C/A	0.607	0.508	1.06 (1.04, 1.08)	1.24E−09	0.700
12q13.12	12	rs11169572	51216890	C/T	0.263	0.381	1.07 (1.05, 1.09)	9.99E−11	0.285
12q24.21	12	rs9634162	115098094	A/G	0.536	0.500	1.06 (1.04, 1.08)	3.29E−08	0.625
14q22.1	14	rs8023022	51366863	T/C	0.456	0.209	1.07 (1.05, 1.09)	1.84E−09	0.727
14q22.2	14	rs17563	54417522	A/G	0.714	0.430	1.06 (1.04, 1.08)	9.30E−09	0.260
15q13.3	15	rs1406389	33009478	T/A	0.716	0.209	1.12 (1.08, 1.15)	1.32E−13	0.186
16q23.2	16	rs12921341	80040583	G/T	0.590	0.468	1.06 (1.04, 1.08)	4.43E−08	0.885
18q21.1	18	rs6507874	46448805	T/C	0.310	0.530	1.15 (1.13, 1.18)	2.66E−36	0.471
18q21.1	18	rs2337107	46459323	T/C	0.328	0.464	1.08 (1.06, 1.10)	1.82E−14	0.036
19q13.11	19	rs73039434	33524919	T/G	0.799	0.945	1.11 (1.07, 1.15)	1.16E−08	0.483
19q13.43	19	rs2305122	59056752	C/G	0.057	0.230	1.10 (1.07, 1.13)	4.66E−10	0.326
20p12.3	20	rs355529	6374388	T/A	0.126	0.300	1.10 (1.08, 1.13)	1.00E−15	0.186
20p12.3	20	rs1015563	6690101	T/C	0.301	0.317	1.09 (1.06, 1.11)	1.14E−14	0.710
20p12.3	20	rs6086208	7765463	T/C	0.330	0.266	1.10 (1.07, 1.12)	3.82E−17	0.015
20p12.3	20	rs2294304	7877079	A/G	0.574	0.989	1.11 (1.08, 1.15)	2.10E−10	0.521
20q13.13	20	rs6066825	47340117	A/G	0.711	0.642	1.07 (1.05, 1.10)	3.41E−11	0.758
20q13.13	20	rs6063515	49056905	G/A	0.478	0.596	1.07 (1.05, 1.10)	2.07E−08	0.438
20q13.13	20	rs6067417	48983697	C/T	0.758	0.579	1.06 (1.04, 1.09)	4.76E−09	0.117
20q13.33	20	rs6121558	60961365	T/C	0.852	0.749	1.11 (1.08, 1.14)	2.16E−13	0.186

*EAS* East Asian population, *EUR* European population, *OR* odds ratio, *95% CI* 95% confidence interval, *GWAS* genome-wide association study, *SNP* single nucleotide polymorphism

^a^Chromosomal position, hg19/GRCh37 build

^b^Risk/reference allele

^c^Risk allele frequency from the 1000 Genomes Project (phase 3) used in this study

^d^Meta-analysis of GWASs in EAS and EUR populations

^e^*P* value for heterogeneity test

### PRS calculation and validation in the independent datasets

Subsequently, we aimed to construct and validate a novel PRS for colorectal cancer risk stratification by incorporating EAS and EUR populations. As shown in Table 2, although the EUR-ancestry PRS showed great discriminatory ability in the EUR population (i.e., CORSA dataset; AUC_crude_ = 0.629, AUC_adjust_ = 0.638), its performance in the EAS population (i.e., JSCRC dataset; AUC_crude_ = 0.511, AUC_adjust_ = 0.510) was limited. Similar results were also found in EAS-ancestry PRS, demonstrating the limited transferability of single-ancestry PRS in other populations.

**Table 2:** Performance evaluation of PRSs derived from different approaches in validation datasets

PRS method	Parameter^a^	N_SNP_	JSCRC GWAS of EAS population			CORSA GWAS of EUR population		
			AUC^b^	OR (95% CI)^c^	*P*^c^	AUC^b^	OR (95% CI)^c^	*P*^c^
GWAS-reported	EUR	140	0.511/0.510	1.04 (0.95, 1.14)	0.432	0.629/0.638	1.65 (1.51, 1.81)	1.49E−28
	EAS	37	0.577/0.580	1.33 (1.21, 1.46)	2.01E−09	0.513/0.506	1.02 (0.94, 1.11)	0.567
C+T	5.00E−08 (0.001)	38	0.569/0.573	1.29 (1.18, 1.42)	6.73E−08	0.579/0.583	1.33 (1.23, 1.45)	1.77E−11
	5.00E−06 (0.001)	88	0.569/0.575	1.30 (1.18, 1.43)	3.30E−08	0.589/0.597	1.39 (1.28, 1.51)	4.02E−14
	5.00E−04 (0.001)	784	0.591/0.597	1.44 (1.31, 1.58)	5.39E−14	0.559/0.567	1.27 (1.16, 1.38)	3.51E−08
	0.05 (0.001)	7128	0.611/0.618	1.52 (1.38, 1.68)	1.52E−17	0.556/0.556	1.23 (1.13, 1.33)	1.65E−06
	5.00E−08 (0.01)	39	0.570/0.573	1.29 (1.18, 1.42)	8.02E−08	0.572/0.574	1.30 (1.20, 1.42)	5.96E−10
	5.00E−06 (0.01)	92	0.571/0.577	1.30 (1.18, 1.42)	4.54E−08	0.583/0.590	1.35 (1.24, 1.47)	4.36E−12
	5.00E−04 (0.01)	854	0.588/0.593	1.42 (1.30, 1.57)	2.62E−13	0.558/0.564	1.25 (1.15, 1.36)	1.04E−07
	0.05 (0.01)	13,989	0.587/0.592	1.37 (1.25, 1.50)	4.12E−11	0.555/0.553	1.21 (1.12, 1.32)	4.89E−06
	5.00E−08 (0.1)	48	0.573/0.577	1.31 (1.20, 1.44)	1.02E−08	0.581/0.581	1.33 (1.22, 1.44)	3.99E−11
	5.00E−06 (0.1)	116	0.579/0.584	1.34 (1.22, 1.47)	7.91E−10	0.592/0.597	1.39 (1.28, 1.51)	3.42E−14
	5.00E−04 (0.1)	992	0.597/0.602	1.46 (1.33, 1.61)	6.02E−15	0.573/0.577	1.31 (1.20, 1.42)	3.22E−10
	0.05 (0.1)	27,032	0.604/0.608	1.52 (1.38, 1.68)	7.05E−18	0.568/0.573	1.29 (1.19, 1.40)	2.61E−09
LDpred	1	883,144	0.611/0.616	1.55 (1.40, 1.70)	8.25E−19	0.560/0.567	1.27 (1.17, 1.38)	2.13E−08
	0.3	883,144	0.612/0.617	1.56 (1.41, 1.71)	3.15E−19	0.560/0.567	1.28 (1.18, 1.39)	8.60E−09
	0.1	883,144	0.614/0.619	1.58 (1.43, 1.74)	3.26E−20	0.567/0.574	1.31 (1.20, 1.42)	4.61E−10
	0.03	883,144	0.621/0.626	1.64 (1.48, 1.80)	6.87E−23	0.586/0.595	1.39 (1.27, 1.51)	6.45E−14
	0.01	883,144	0.633/0.638	1.68 (1.52, 1.85)	7.86E−25	0.602/0.608	1.47 (1.35, 1.60)	2.04E−18
	0.003	883,144	0.495/0.491	0.98 (0.89, 1.07)	0.627	0.514/0.513	1.02 (0.94, 1.11)	0.663
	0.001	883,144	0.508/0.509	1.04 (0.95, 1.14)	0.436	0.491/0.490	0.95 (0.88, 1.04)	0.257
	3.00E−04	883,144	0.499/0.499	0.99 (0.91, 1.09)	0.885	0.493/0.491	0.98 (0.91, 1.07)	0.704
	1.00E−04	883,144	0.487/0.489	0.94 (0.86, 1.03)	0.202	0.510/0.508	1.04 (0.96, 1.13)	0.343
	3.00E−05	883,144	0.494/0.498	0.98 (0.89, 1.07)	0.670	0.501/0.507	1.03 (0.95, 1.12)	0.464
	1.00E−05	883,144	0.480/0.482	0.95 (0.87, 1.04)	0.277	0.505/0.500	1.02 (0.94, 1.11)	0.653
Lassosum	Optimal	5984	0.606/0.610	1.51 (1.37, 1.66)	4.53E−17	0.601/0.605	1.45 (1.33, 1.58)	2.12E−17
LDpred2	Auto	890,687	0.570/0.573	1.30 (1.19, 1.43)	2.36E−08	0.557/0.563	1.24 (1.14, 1.35)	3.19E−07
**PRS-CSx**^**#**^	**Auto**	**1,145,689**	**0.639/0.646**	**1.73 (1.56, 1.91)**	**7.19E−27**	**0.602/0.608**	**1.48 (1.36, 1.62)**	**5.18E−19**

*EAS* East Asian population, *EUR* European population, *PRS* polygenic risk score, *C+T* Clumping and *P* value thresholding, *AUC* area under the receiver operating characteristics curve, *95% CI* 95% confidence interval, *OR* odds ratio, *SD* standard deviation, *GWAS* genome-wide association study, *SNP* single nucleotide polymorphism, *CORSA* Colorectal Cancer Study of Austria

^a^Parameter for SNP selection: population for GWAS-reported variants; *P* value (LD *r*^2^) for C+T method; fraction for LDpred method; optimal parameter for lassosum method, auto parameter for LDpred2, and PRS-CSx methods

^b^Crude AUC/covariates-adjusted AUC

^c^OR (95% CI) per SD, derived from logistic model with the adjustment of sex, age, and principal components

^#^The optimal PRS was highlighted in bold

Among the 26 developed EAS-EUR PRSs, twenty were significantly associated with an increased risk of developing colorectal cancer in the JSCRC GWAS of EAS ancestry [OR per standard deviation (SD) increase ranged from 1.29 (*P* = 8.02 × 10^−8^) for C+T (*P* value and LD *r*^2^: 5 × 10^−8^ and 0.01) to 1.73 (*P* = 7.19 × 10^−27^) for PRS-CSx], as well as in the CORSA GWAS of EUR ancestry [OR per SD ranged from 1.21 (*P* = 4.89 × 10^−6^) for C+T (*P* value and LD *r*^2^: 0.05 and 0.01) to 1.48 (*P* = 5.18 × 10^−19^) for PRS-CSx; Table 2]. Notably, the PRS-CSx approach-based PRS that harbored genome-wide 1,145,689 SNPs (defined as PRS_CSx_) achieved the optimal discriminatory ability for distinguishing cases from healthy controls in both validation datasets (JSCRC dataset: AUC_crude_ = 0.639, AUC_adjust_ = 0.646; Additional file 1: Fig. S4; CORSA dataset: AUC_crude_ = 0.602, AUC_adjust_ = 0.608; Additional file 1: Fig. S5). Especially, when compared with known variant-derived PRS, the PRS_CSx_ showed better predictive performance in the EAS population than both EUR-ancestry (AUC_adjust_: 0.646 vs. 0.510) and EAS-ancestry PRSs (AUC_adjust_: 0.646 vs. 0.580), although it had a marginally weaker predictive ability in EUR population than EUR-ancestry PRS (AUC_adjust_: 0.608 vs. 0.638).

### PRS test in the UK Biobank cohort

**Fig. 2:**
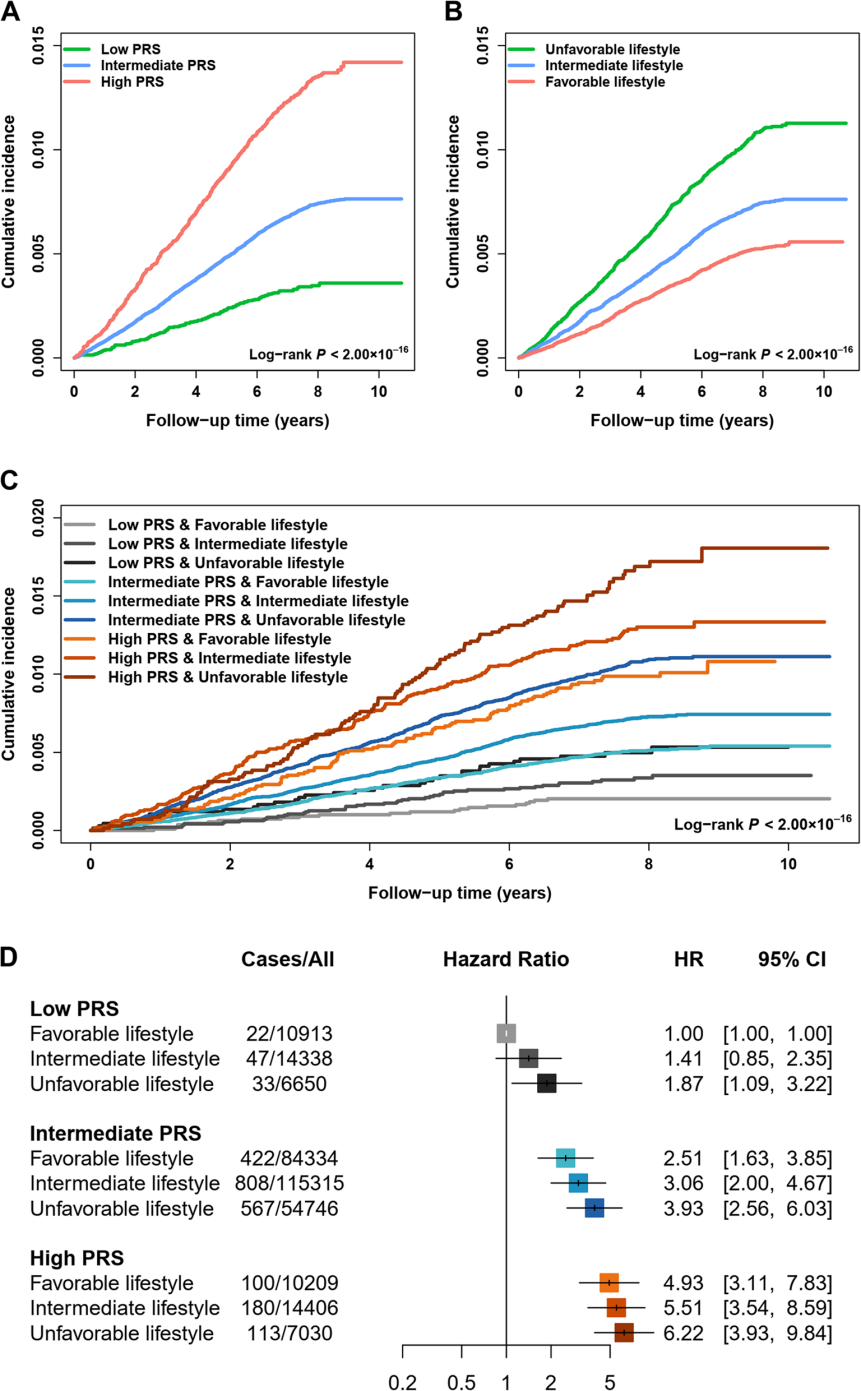
The cumulative risk of developing colorectal cancer according to the PRS and lifestyle score in the UK Biobank cohort. **A** Cumulative incidence of colorectal cancer in the low, intermediate, and high PRS groups. **B** Cumulative incidence of colorectal cancer in unfavorable, intermediate, and favorable lifestyle groups. **C** Cumulative incidence of colorectal cancer stratified by different levels of PRS and lifestyle score. **D** The associations of PRS and lifestyle score with incident colorectal cancer. The HR and 95% CI were derived from the Cox regression model with the adjustment of sex, age, center, and first 10 principal components. PRS, polygenic risk score; HR, hazard ratio; 95% CI, 95% confidence intervals

We further evaluated the performance of the optimal PRS_CSx_ for colorectal cancer risk prediction in the UK Biobank cohort, in which 2621 colorectal cancer cases among 355,543 individuals were confirmed during a median follow-up of 7.88 years. As expected, colorectal cancer cases had a higher PRS_CSx_ value than those without colorectal cancer [HR = 1.42, 95% CI = 1.37 to 1.48 per SD increase, *P* = 3.53 × 10^−72^, Additional file 1: Table S7; *P*_Wilcoxon_ < 2 × 10^−16^; Additional file 1: Fig. S6A]. Importantly, PRS_CSx_ had a stable discriminatory ability with an AUC of 0.595 (for crude AUC) and 0.597 (for covariates-adjusted AUC; Additional file 1: Fig. S6B), similar with that in the validation dataset of EUR ancestry. Notably, there was a dose-response effect of PRS_CSx_ on developing colorectal cancer at both decile classification (*P*_trend_ = 1.57 × 10^−56^; Additional file 1: Fig. S6C) and three-category classification (intermediate vs. low: HR = 2.11, 95% CI = 1.76 to 2.54, *P* = 1.30 × 10^−15^; high vs. low: HR = 3.88, 95% CI = 3.18 to 4.74, *P* = 2.82 × 10^−40^; *P*_trend_ = 8.15 × 10^−53^; Additional file 1: Table S7; log-rank *P* < 2 × 10^−16^; Fig. 2A). Besides, we observed similar findings underlying the sensitivity analyses (Additional file 1: Table S8).

### Evaluation of the benefit of adherence to a healthy lifestyle stratified by genetic risk

In the UK Biobank cohort, several healthy lifestyle factors were associated with a decreased risk of colorectal cancer; for example, compared to smokers, non-smokers had a 0.18-fold reduced risk of developing colorectal cancer (OR = 0.82, *P* = 3.58 × 10^−7^; Additional file 1: Table S4). Furthermore, we noticed a significantly protective effect of combined lifestyle score in a dose-response manner on colorectal cancer development at both continuous levels (HR = 0.90, 95% CI = 0.88 to 0.93 per lifestyle score increase, *P* = 3.39 × 10^−12^; Additional file 1: Table S9) and stratified levels (intermediate vs. unfavorable: HR = 0.79, 95% CI = 0.72 to 0.87, *P* = 2.86 × 10^−6^; favorable vs. unfavorable: HR = 0.65, 95% CI = 0.58 to 0.74, *P* = 2.56 × 10^−12^; *P*_trend_ = 1.92 × 10^−12^; log-rank *P* < 2 × 10^−16^; Fig. 2B). Similar findings were observed in the sensitivity analyses (Additional file 1: Table S10). Intriguingly, there was an inverse relationship between the PRS_CSx_ and several lifestyle factors (*P*_Wilcoxon_ < 0.05; Additional file 1: Fig. S7A) or the lifestyle score (*P*_Kruskal-Wallis_ = 1.60 × 10^−8^; *P*_chi-square_ = 9.83 × 10^−7^; Additional file 1: Fig. S7B-C), but their effects on colorectal cancer risk were not mutually influenced (Additional file 1: Tables S7-10).

Therefore, we further evaluated the joint effect of genetic and lifestyle factors on the risk for incident colorectal cancer. As expected, there was a notable dose-response manner on increasing colorectal cancer risk as PRS_CSx_ increased and lifestyle score decreased (trend to unfavorable lifestyle) (log-rank *P* < 2 × 10^−16^; Fig. 2C, D), but no multiplicative interaction between genetic risk and lifestyle score was observed (*P*_interaction_ = 0.539). Interestingly, when stratifying individuals by PRS_CSx_ categories, we observed that a healthy lifestyle could still be significantly associated with a reduced risk of developing colorectal cancer broadly, regardless of the genetic risk effect (low: *P*_trend_ = 0.043, intermediate: *P*_trend_ = 7.18 × 10^−11^, high: *P*_trend_ = 0.077; Table 3). Similar trends were found in the sensitivity analyses (Additional file 1: Table S11).

**Table 3:** Cumulative risk of developing colorectal cancer according to different levels of PRS and lifestyle score in the UK Biobank cohort

PRS	Lifestyle	Cases/all	Incidence proportion (95% CI)	HR (95% CI)^a^	*P*^a^	*P*_trend_	5-year absolute risk (reduction)^b^
Low	Unfavorable	33/6650	0.50% (0.34, 0.70)	1.00 (reference)			0.28% (reference)
	Intermediate	47/14,338	0.33% (0.24, 0.44)	0.76 (0.48, 1.21)	0.248		0.19% (0.09%)
	Favorable	22/10,913	0.20% (0.13, 0.31)	0.55 (0.31, 0.98)	0.044	0.043	0.14% (0.14%)
Intermediate	Unfavorable	567/54,746	1.04% (0.95, 1.12)	1.00 (reference)			0.61% (reference)
	Intermediate	808/115,315	0.70% (0.65, 0.75)	0.78 (0.70, 0.87)	9.18E−06		0.42% (0.19%)
	Favorable	422/84,334	0.50% (0.45, 0.55)	0.64 (0.56, 0.73)	9.92E−11	7.18E−11	0.31% (0.31%)
High	Unfavorable	113/7030	1.61% (1.32, 1.93)	1.00 (reference)			1.07% (reference)
	Intermediate	180/14,406	1.25% (1.07, 1.45)	0.87 (0.68, 1.11)	0.255		0.75% (0.32%)
	Favorable	100/10,209	0.98% (0.80, 1.19)	0.77 (0.58, 1.03)	0.078	0.077	0.54% (0.53%)

*PRS* polygenic risk score, *HR* hazard ratio, *95% CI* 95% confidence intervals

^a^Derived from Cox regression model with the adjustment of sex, age, center, and first 10 principal components

^b^Mean value (reduction) of 5-year absolute risk

### Estimation of 5-year absolute risk

**Fig. 3:**
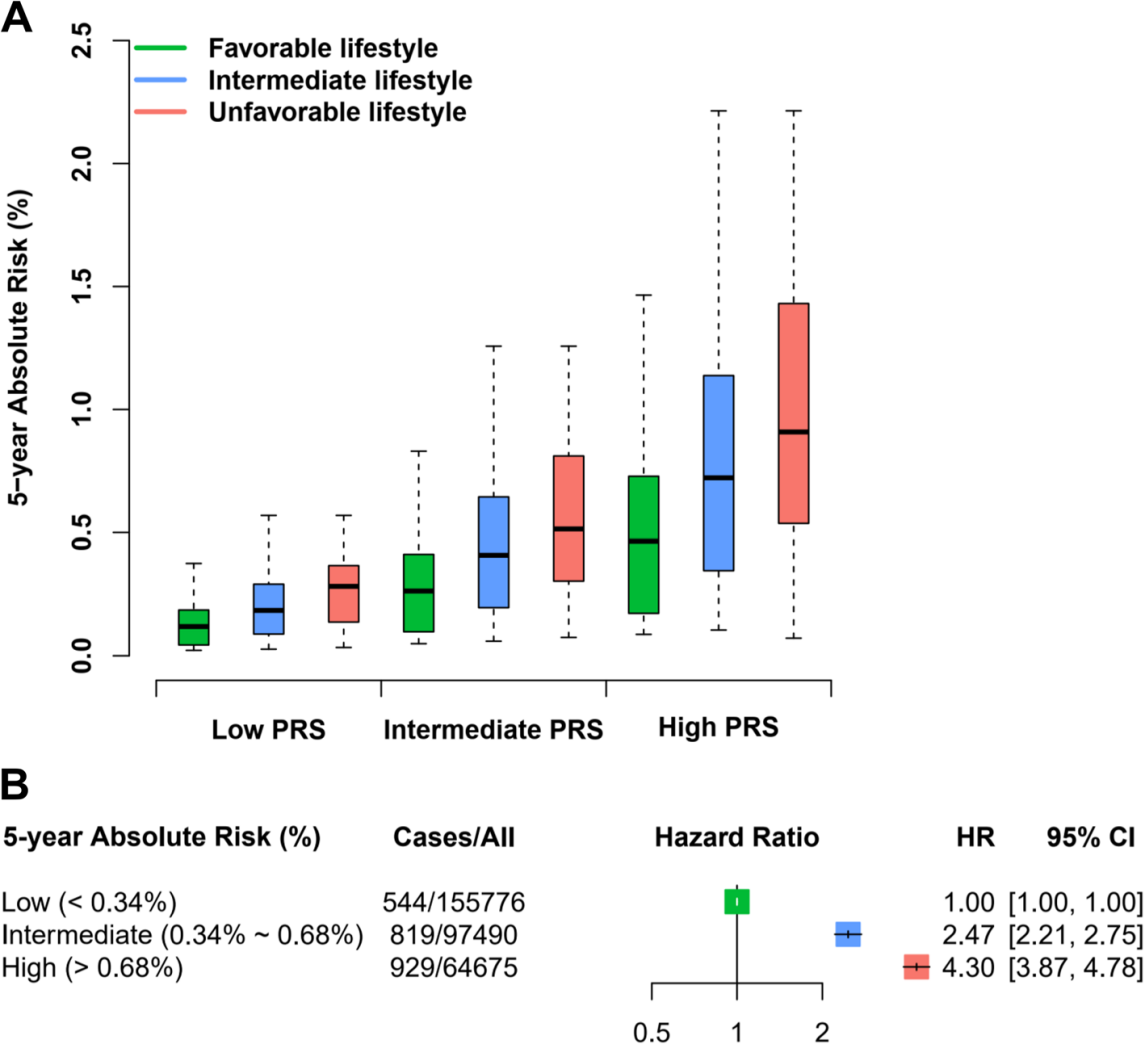
Estimation of 5-year absolute risk for colorectal cancer in the UK Biobank cohort. **A** The 5-year absolute risk of developing colorectal cancer defined by different levels of PRS and lifestyle score. **B** The associations between different levels of 5-year absolute risk and incident colorectal cancer. The HR and 95% CI were derived from the Cox regression model with the adjustment of center and first 10 principal components. PRS, polygenic risk score; HR, hazard ratio; 95% CI, 95% confidence intervals

Subsequently, we estimated the 5-year absolute risk of developing colorectal cancer using a combination of genetic and lifestyle factors and observed that colorectal cancer patients had a higher 5-year absolute risk than those without colorectal cancer (*P*_Wilcoxon_ < 2 × 10^−16^; Additional file 1: Fig. S8A). Especially when stratified by age group, a higher 5-year absolute risk was observed in individuals carrying a high genetic risk or an unfavorable lifestyle (*P*_Kruskal-Wallis_ < 2 × 10^−16^; Additional file 1: Fig. S8B-C). Furthermore, in the stratification by genetic risk (Table 3 and Fig. 3A), there was a significant risk reduction in individuals with a low PRS and a favorable lifestyle (risk = 0.14%, reduction = 0.14%) compared with those with a low PRS but an unfavorable lifestyle (risk = 0.28%), and among individuals with a high PRS, the risk of an unfavorable lifestyle increased to 1.07%, which could be reduced to 0.54% among those with a favorable lifestyle (reduction = 0.53%).

### Construction of ColoRectal Cancer Risk Prediction System (CRC-RPS)

Furthermore, we stratified the risk population according to the median value (0.34%; as a reference threshold) and two times the threshold (0.68%) of 5-year absolute risk among individuals without colorectal cancer, which was defined as low (< 0.34%), intermediate (0.34 to 0.68%) and high risk (> 0.68%). As expected, both intermediate- and high-risk populations had a higher risk of developing colorectal cancer than the low-risk population (intermediate: HR = 2.47, 95% CI = 2.21 to 2.75; high: HR = 4.30, 95% CI = 3.87 to 4.78; Fig. 3B). To friendly apply our findings, we developed a colorectal cancer risk prediction web server, CRC-RPS, to help users estimate their 5-year absolute risk of developing colorectal cancer by combining genetic and lifestyle factors (http://njmu-edu.cn:3838/CRC-RPS/). In brief, users can easily input their sex, age, and lifestyle information along with the genotypes of 1.15 million SNPs to obtain an estimated 5-year absolute risk and the assigned risk-population group. For example, a user with a predicted 0.2% of 5-year absolute risk was grouped as low risk of developing colorectal cancer.

## Discussion

In the present study, we comprehensively constructed several sets of EAS-EUR PRSs based on the large-scale GWAS data of colorectal cancer across EAS and EUR populations and subsequently found a solid PRS framework (i.e., PRS_CSx_) derived from genome-wide SNPs, independent of individual lifestyle, for stratifying the risk populations of developing colorectal cancer evidenced by independent validation datasets and a longitudinal cohort. Importantly, even though there was diversity in genetic risk, adherence to a healthy lifestyle behavior could consistently reduce the risk of developing colorectal cancer.

In recent decades, convincing evidence has emerged suggesting that identifying high-risk individuals can enable enhanced screening and the application of other interventions, thereby reducing the incidence of colorectal cancer [35]. Therefore, researchers have paid more attention to the clinical use of PRS, by determining whether it can stratify populations into subgroups with a distinct risk of developing diseases for early interventions [8, 36]. To date, multiple PRSs have been constructed and confirmed to have a discriminatory ability in distinguishing colorectal cancer cases from healthy controls [9, 10, 37]. However, most PRSs were derived from individuals of EUR ancestry, which might limit their application in other ethnic populations. Cumulative evidence has demonstrated that, when applying the PRS models trained with EUR individuals to other ethnic populations, there were less accurate compared to EUR populations [11, 38]. In particular, Thomas et al. found that the PRS model of colorectal cancer derived from 120,184 subjects of EUR ancestry performed worse for Asians, Hispanics, and African Americans than for Europeans [10]. These findings highlighted the need to reconsider the model performance when applying PRS to non-European ancestry and bolstered the rationale for trans-ancestry PRS in diverse populations. Here, we built a novel PRS_CSx_ across EAS and EUR populations and validated that this PRS could significantly predict the risk of developing colorectal cancer in two ethnic groups; importantly, the high PRS group could be used in colorectal cancer screening for personalized prevention.

Although the performance of our PRS in the EUR population (e.g., CORSA dataset) is substantially lower than previous EUR-ancestry PRSs (e.g., Thomas et al.’s genome-wide PRS) [10], our aim was to improve the clinical utility of PRS in multiple ethnic groups, especially for non-EUR (e.g., EAS) populations. As evidenced in a recent trans-ancestry PRS study, when the target population was EUR population, the improvement of multi-ancestry PRS over EUR-ancestry PRS was limited; however, when predicting into EAS populations, multi-ancestry PRS clearly outperformed EUR-ancestry PRS [31], which was also found in our study. Therefore, the advantage of our PRS compared to EUR-ancestry PRSs should be further validated in independent EAS longitudinal cohorts.

A healthy lifestyle has been known to be associated with a decreased risk of colorectal cancer. For instance, Kirkegaard et al. found that 23% of colorectal cancer cases might be caused by a lack of adherence to five lifestyle recommendations in a prospective Danish cohort study with 55,487 participants [39]. In our study, another important finding was that the detrimental effect of high genetic risk on incident colorectal cancer could be largely attenuated by adherence to a healthy lifestyle, which was consistent with previous findings [13, 32, 40]. Moreover, although the 5-year absolute risk associated with adherence to a healthy lifestyle was greatest in the group at high genetic risk, our results still emphasize the notion that the public senses of a healthy lifestyle in the whole population will lead to an evident reduction in colorectal cancer risk.

This study has several strengths. First, to our knowledge, this is the first study to develop an EAS-EUR PRS with a sufficient sample size, followed by the performance evaluation on incident colorectal cancer risk via external case-control studies and prospective cohort. This study provided further genetic information supporting the contribution of germline variation to ancestry disparity in the development of colorectal cancer. Second, we constructed a user-friendly web server to help generate a customized estimate of risk for developing colorectal cancer, for use as an early screening method. Nevertheless, we acknowledge several limitations. First, we need to validate the predictive ability of this novel PRS in an independent EAS longitudinal cohort with sufficient samples. Second, we currently focus on EAS and EUR populations in this study, and other populations (e.g., African Americans and Hispanics) need to be included in future work. Third, the limited model performance in the EUR population needs to be further improved using a larger sample size in the training set, as well as more sophisticated trans-ancestry PRS methods.

## Conclusions

In conclusion, we applied an EAS-EUR combined approach to construct a PRS framework derived from genome-wide SNPs that can effectively predict colorectal cancer risk, which reduced the gap in genetic risk prediction between diverse populations. Importantly, these findings also provided further evidence that a healthy lifestyle can attenuate the genetic impact on incident colorectal cancer.

## Supplementary Information

**Additional file 1: Table S1.** Basic characteristics of colorectal cancer GWASs. **Table S2**. Basic characteristics of the UK Biobank cohort. **Table S3**. Summary of 37 colorectal cancer GWAS-reported SNPs in East Asian. **Table S4**. Summary of eight lifestyle factors in the UK Biobank cohort. **Table S5**. Summary of one novel EAS-EUR conditionally independent variant at known colorectal cancer risk loci. **Table S6**. Functional annotations of one novel colorectal cancer risk locus. **Table S7**. The association of PRS with colorectal cancer risk in the UK Biobank. **Table S8**. Sensitivity analyses for the association of PRS with colorectal cancer risk in the UK Biobank cohort. **Table S9**. The association of lifestyle score with colorectal cancer risk in the UK Biobank cohort. **Table S10**. Sensitivity analyses for the association of lifestyle score with colorectal cancer risk in the UK Biobank cohort. **Table S11**. Sensitivity analyses for cumulative risk of developing colorectal cancer according to different levels of PRS and lifestyle score in the UK Biobank cohort. **Fig. S1**. Principal component analysis based on the colorectal cancer GWAS subjects and 1000 Genomes Project populations. **Fig. S2**. Quantile-quantile plot and genomic inflation factor for the association with colorectal cancer risk in the meta-analysis of EAS-EUR GWASs. **Fig. S3**. Manhattan plot from colorectal cancer EAS-EUR GWAS meta-analysis. **Fig. S4**. The association of PRS_CSx_ with incident colorectal cancer in the JSCRC GWAS dataset. **Fig. S5**. The association of PRS_CSx_ with incident colorectal cancer in the CORSA GWAS dataset. **Fig. S6**. The association of PRS with incident colorectal cancer in the UK Biobank cohort. **Fig. S7**. The association of PRS with lifestyle factors in the UK Biobank cohort. **Fig. S8**. Distribution of 5-year absolute risk of developing colorectal cancer in the UK Biobank cohort.

## Data Availability

BBJ colorectal cancer GWAS summary statistics are publicly available on the JENGER website (http://jenger.riken.jp/en/result). GWAS summary statistics from the GECCO study are available on the database of Genotypes and Phenotypes (dbGaP; Study Accession: phs001315.v1.p1; phs001415.v1.p1 and phs001078.v1.p1). GWAS summary statistics from the PLCO study are publicly available on the PLCOjs website (https://episphere.github.io/plco/#). Individual-level data from the UK Biobank cohort are available through the UK Biobank (https://www.ukbiobank.ac.uk/) application. The genotype data from the Chinese population cannot be submitted to publicly available databases because the ethical approval did not permit the sharing of raw genotype data. But the data can be shared upon reasonable request to the corresponding author in accordance with the Chinese genomic data sharing policy. The SNP effect size estimates for the PRS_CSx_ are available at http://njmu-edu.cn:3838/CRC-RPS/ and are deposited in the PGS Catalog (https://www.pgscatalog.org; PGS ID: PGS003395).
