# Supplementary material for: Risk assessment for colorectal cancer via polygenic risk score and lifestyle exposure: a large-scale association study of East Asian and European populations
Source: Genome Med. 2023 Jan 24;15:4. doi: 10.1186/s13073-023-01156-9 (PMC9875451; doi:10.1186/s13073-023-01156-9)
Supplement: Supplementary file 1 — Additional file 1: Table S1. Basic characteristics of colorectal cancer GWASs. Table S2. Basic characteristics of the UK Biobank cohort. Table S3. Summary of 37 colorectal cancer GWAS-reported SNPs in East Asian. Table S4. Summary of eight lifestyle factors in the UK Biobank cohort. Table S5. Summary of one novel EAS-EUR conditionally independent variant at known colorectal cancer risk loci. Table S6. Functional annotations of one novel colorectal cancer risk locus. Table S7. The association of PRS with colorectal cancer risk in the UK Biobank. Table S8. Sensitivity analyses for the association of PRS with colorectal cancer risk in the UK Biobank cohort. Table S9. The association of lifestyle score with colorectal cancer risk in the UK Biobank cohort. Table S10. Sensitivity analyses for the association of lifestyle score with colorectal cancer risk in the UK Biobank cohort. Table S11. Sensitivity analyses for cumulative risk of developing colorectal cancer according to different levels of PRS and lifestyle score in the UK Biobank cohort. Fig. S1. Principal component analysis based on the colorectal cancer GWAS subjects and 1000 Genomes Project populations. Fig. S2. Quantile-quantile plot and genomic inflation factor for the association with colorectal cancer risk in the meta-analysis of EAS-EUR GWASs. Fig. S3. Manhattan plot from colorectal cancer EAS-EUR GWAS meta-analysis. Fig. S4. The association of PRSCSx with incident colorectal cancer in the JSCRC GWAS dataset. Fig. S5. The association of PRSCSx with incident colorectal cancer in the CORSA GWAS dataset. Fig. S6. The association of PRS with incident colorectal cancer in the UK Biobank cohort. Fig. S7. The association of PRS with lifestyle factors in the UK Biobank cohort. Fig. S8. Distribution of 5-year absolute risk of developing colorectal cancer in the UK Biobank cohort. [file 13073_2023_1156_MOESM1_ESM.pdf]

Supplementary Materials

Study subjects

Case-control studies

East Asian (EAS) of Chinese population

NJCRC GWAS

The colorectal cancer cases were recruited from the Cancer Center of Nanjing Medical University, and the cancer-free controls were from the same districts of Nanjing. The population details of the NJCRC GWAS have been described in previous studies [1, 2].

BJCRC GWAS

All participants were recruited from Beijing, colorectal cancer cases were recruited from local hospitals and had pathologically proven disease. Cancer-free control subjects were recruited in local hospitals for individuals receiving routine physical examinations or in the communities for those participating screening of non-communicable diseases. The population details have been described in previous study [3].

SHCRC GWAS

The colorectal cancer cases were recruited from the Fudan University Shanghai Cancer Center (FUSCC), and the cancer-free controls were from the same districts of Shanghai. The population details about the cases included in SHCRC GWAS have been described in previous study [4].

ZJCRC GWAS

The individuals of ZJCRC GWAS cases were derived from the Jiashan Institute of Cancer Prevention and Treatment, and the Second Affiliated Hospital of Zhejiang University School of Medicine. The cancer-free controls were selected from individuals receiving routine physical examination at hospitals or those participating in community screening for non-communicable diseases in Zhejiang province.

JSCRC GWAS

The confirmed cases were consecutively recruited from hospitals in Jiangsu province, China. The cancer-free control subjects were selected from individuals receiving routine physical examination at hospitals or those participating in community screening for non-communicable diseases in Jiangsu province.

For all Chinese GWASs, we used a uniform individual quality control protocol to filter the samples as follows: (i) call rate < 95%; (ii) gender discrepancies; (iii) unexpected duplicates or probable relatives based on pairwise identity by descent (PI_HAT > 0.25); and (iv) population stratification outliers based on EIGENSTRAT.

EAS of Japanese population

All case samples of the Japanese GWAS were collected in the BioBank Japan Project (BBJ), which is a biobank that recruited approximately 200,000 patients with a diagnosis of at least one of 47 diseases in Japan. The healthy controls were from several population-based prospective cohorts and participants without related diagnoses in the BBJ.

European (EUR) population

Genetics and Epidemiology of Colorectal Cancer Consortium (GECCO)

We collected GWAS datasets of GECCO consortia from the database of Genotypes and Phenotypes (dbGaP, phs001315.v1.p1; phs001415.v1.p1 and phs001078.v1.p1). After individual-level quality control: (i) unexpected duplicates or probable relatives based on pairwise identity by descent (PI_HAT > 0.25); (ii) restricted to individuals of EUR ancestry; and (iii) excluded participants recruited from Prostate, Lung, Colorectal and Ovarian (PLCO) cancer screening trial and Colorectal Cancer Study of Austria (CORSA) dataset, a total of 21,608 cases and 20,278 cancer-free controls were retained for analysis.

PLCO

The PLCO cancer screening trial is a population-based cohort study that aims to evaluate the accuracy and reliability of screening methods for prostate, lung, colorectal and ovarian cancer, among which 154,897 individuals aged 55–74 years were randomly recruited from 10 United States research centers from 1993 to 2001.

CORSA

In the CORSA study, more than 13,000 Caucasian participants have been recruited within the province-wide screening project "Burgenland Prevention Trial of Colorectal Disease with Immunological Testing" (B-PREDICT) since 2003. All inhabitants of the Austrian province Burgenland aged between 40 and 80 years are annually invited to participate in fecal immunochemical testing and haemoccult positive screening participants are invited for colonoscopy. CORSA participants were recruited from the four KRAGES hospitals in Burgenland, Austria, and additionally, at the Medical University of Vienna (Department of Surgery), the Viennese hospitals

"Rudolfstiftung" and the "Sozialmedizinisches Zentrum Sud", and at the Medical University of Graz (Department of Internal Medicine). We accessed the CORSA genotype data from dbGaP (phs001415.v1.p1); after individual-level quality control: (i) unexpected duplicates or probable relatives based on pairwise identity by descent (PI_HAT > 0.25); and (ii) restricted to individuals of EUR ancestry, a total of 1,289 cases and 1,284 controls were retained for analysis.

Longitudinal cohort of UK Biobank

The UK Biobank cohort is a prospective, population-based study, which recruited 502,528 adults aged 40-69 years from the general population between April 2006 and December 2010. Participants visited one of 22 assessment centers across England, Scotland and Wales, where they completed touchscreen and nurse-led questionnaires, and provided biological samples.

After individual-level quality control: (i) removed individuals with prevalent cancer [except non-melanoma skin cancer, based on the International Classification of Diseases, 10th revision (ICD-10, C44)] at baseline; (ii) sex discordance; (iii) outliers for genotype missingness or excess heterozygosity; (iv) retained unrelated participants; (v) restricted to "white British" participants individuals of EUR ancestry and (vi) removed individuals who decided not to participate in this program, a total of 355,543 participants were retained for analysis.

Genotyping and imputation

Case-control GWAS study

EAS of Chinese population

Genomic DNA was derived from EDTA-venous blood by using the Qiagen Blood Kit (Qiagen). Genotyping was conducted using Illumina Human Omni ZhongHua Bead Chips for NJCRC GWAS, Illumina Infinium Global Screening Assay (GSA) for SHCRC GWAS, Affymetrix Axiom Genome-Wide CHB1 and CHB2 Arrays for BJCRC GWAS, and Illumina Asian Screening Array (ASA) for JSCRC and ZJCRC GWASs. Furthermore, we imputed the non-genotyped SNPs based on the 1000 Genomes Project (Phase 1 or Phase 3) using IMPUTE2. GTOOL was used to convert imputed data into PLINK format with a threshold of 0.9.

EAS of Japanese population

The samples were genotyped with the Illumina HumanOmniExpressExome BeadChip or a combination of the Illumina HumanOmniExpress and HumanExome BeadChips. The genotyping data were then imputed using SHAPEIT2 and minimac3 based on the reference panels of the 1000 Genomes Project (Phase 3). More details have been reported previously [5].

EUR population

GECCO and *CORSA*

Genomic DNA extracted from the blood samples was genotyped using Infinium OncoArray-500K array (dbGaP accession: phs001415.v1.p1), Illumina HumanOmniExpressExome-8v1–2 array (dbGaP accession: phs001315.v1.p1), and Illumina HumanCytoSNP-12v2 or HumanOmniExpress (dbGaP accession: phs001078.v1.p1). More details can be found in previous studies [6, 7]. Imputations

of dbGap studies were performed using the 1000 Genomes Project (Phase 1) or Haplotype Reference Consortium (HRC) reference panel.

PLCO

Sequential blood samples were collected from participants assigned to the screening arm. Ninety-three percent of participants assigned to the screening arm provided a baseline blood sample. In the observational (control) arm, buccal cells were collected via mail using the “swish-and-spit” protocol, and the participation rate was 65%. The detailed information can be found in our previous study [8]. A more detailed description of the PLCO study is available online (http://dcp.cancer.gov/plco). We obtained the GWAS summary statistics from the PLCOjs website (https://exploregwas.cancer.gov/plco-atlas; dbGaP accession: phs001286.v2.p2), of which the non-genotyped SNPs were imputed based on haplotypes derived from TOPMED reference panel 5b using Michigan Imputation Server (https://imputationserver.sph.umich.edu).

Longitudinal cohort of UK Biobank

All samples were genotyped using the UK BiLEVE Axiom Array (807,411 markers tested for 49,950 participants) or UK Biobank Axiom Array (825,927 markers tested for 438,427 participants) by Affymetrix. The genotyping data were imputed using SHAPEIT3 and IMPUTE3 based on the reference panels of HRC, UK10K and 1000 Genomes Project (Phase 3). The study protocol and information about data access are available online (http://www.ukbiobank.ac.uk/wp-content/uploads/2011/11/UK-Biobank-Protocol.pdf) and more details of the recruitment and study design have been published in previous

studies [9, 10].

For each GWAS, the imputed SNPs located within autosomal chromosomes were removed if they had (i) minor allele frequency (MAF) < 0.01; (ii) call rate < 95%; (iii) Hardy-Weinberg equilibrium (HWE) *P*-value in controls < 1×10^{-6} and (iv) information metric (info score) < 0.3.

Functional annotation and expression quantitative trait loci (eQTL) analysis

We performed functional annotation for novel loci based on HaploReg v4.1 (http://archive.broadinstitute.org/mammals/haploreg/haploreg.php), RegulomeDB (http://regulome.stanford.edu/) and SNPinfo Web Server (http://snpinfo.niehs.nih.gov/). Besides, to examine predicted functional impact, we annotated variants with the CADD score (Phred scores >20 predicted as deleterious, https://cadd.gs.washington.edu/score).

Furthermore, we performed eQTL analysis between novel loci and their nearby genes (within ±1 Mb region) using the data from the Genotype-Tissue Expression (GTEx), in which normal Colon-Sigmoid tissues and Colon-Transverse tissues were included in the analysis, the details can be found in the GTEx website (https://www.gtexportal.org/home/).

Calculation of polygenic risk score (PRS)

Known GWAS-identified variant-based PRS

We used colorectal cancer GWAS-identified variants for the construction of

EAS-ancestry and EUR-ancestry PRSs. For the EUR-ancestry PRS, we used the previously reported 140 independent variants for calculation [11]. For the EAS-ancestry PRS, a total of 37 independent SNPs (LD $r^2 < 0.1$; **Additional File 1: Table S3**) were collected for construction. The weights of the two PRSs were derived from previous GWASs.

Clumping and P value thresholding

The clumping and *P* value thresholding approach, a classic PRS method, commonly known as C+T method, which constructed PRSs based on a subset of partially independent (i.e., clumped) SNPs exceeding a specific GWAS association *P* value threshold [12]. Based on the summary statistics of EAS-EUR meta-analysis, we used PLINK software (version 1.90) to determine three subsets of variants, where we set the region size to be 500 kb and the linkage disequilibrium (LD) r^2 to 0.001, 0.01 and 0.1, with different *P* value thresholds (i.e., 5×10^{-8}, 5×10^{-6}, 5×10^{-4} and 0.05).

LDpred

LDpred is a Bayesian genome-wide genetic risk prediction method by calculating a posterior mean effect size for each variant based on a prior and subsequent shrinkage based on the LD among SNPs, implemented in the LDpred python software [13]. First, we restricted the variants to the HapMap3 panel to circumvent the non-convergence issue from training on summary statistics of EAS-EUR meta-analysis. Subsequently, the fraction of causal (i.e., non-zero effect sizes) variants was set as 1, 0.3, 0.1, 0.03, 0.01, 0.003, 0.001, 3×10^{-4}, 1×10^{-4}, 3×10^{-5} and 1×10^{-5}, to construct 11 candidate PRSs.

Lassosum

Lassosum was used to construct PRS based on a penalized regression framework taking into account LD information from a reference panel, which was implemented with the R package *lassosum* [14]. Based on the summary statistics of EAS-EUR meta-analysis, we selected the optimal elastic net tuning parameters (s and lambda) validated in the target dataset (i.e., CORSA GWAS) to define the lassosum-based PRS.

LDpred2

LDpred2 is an extension of the LDpred method which derives the PRS based on summary statistics and a matrix of correlation between genetic variants, which was implemented with the R package *bigsnpr* [15]. We used the LDpred-auto model (i.e., automatically estimates sparsity P and heritability h^2 from the reference panel) to construct PRS, based on the EAS-EUR meta-analyzed summary statistics that were restricted in HapMap3 panel.

PRS-CSx

PRS-CSx, a recently proposed Bayesian polygenic modeling method, has been demonstrated to be useful for constructing trans-ancestry PRS [16, 17]. We applied PRS-CSx python software to jointly model GWAS summary statistics in HapMap3 panel across two populations using a shared continuous shrinkage prior, which enabled more accurate effect size estimation. We used the pre-computed 1000 Genomes Project reference panels, and a fully Bayesian algorithm for model fitting, which automatically learned all model parameters without the need for

hyper-parameter tuning. Population-specific posterior effect size estimates were further combined using an inverse-variance-weighted meta-analysis within the Gibbs sampler (--meta).

Construction of lifestyle score

The score for each lifestyle factor was created by evaluating whether they met (i.e., 1) or not met (i.e., 0) the guidelines as follows (**Additional File 1: Table S4**): BMI, weight (kg) / height (m^2), was classified into healthy behavior category if they were within 18.5–24.9. Tobacco smoking and alcohol consumption with "Never" status were considered the healthy category. WHR, waist-to-hip ratio, met a healthy lifestyle requirement if it <0.90 for men and <0.85 for women. Participants were classified as having met current physical activity recommendations if they reported 6–7 days per week of moderate activity and 3–5 days per week of vigorous activity, or 6–7 days per week of vigorous activity. Sedentary time was calculated based on the sum of the time watching television and using computer. Less than 3 hours per day was considered healthy. For red and processed meat, we summed the frequencies for beef, pork, lamb/mutton, and processed meat, using the following coding: "Never" = 0, "Less than once a week" = 0.5, "Once a week" = 1, "2-4 times a week" = 3, "5-6 times a week" = 5.5 and "Once or more daily" = 7. We classified < 4 times per week into healthy behavior category. For fruit, participants were asked to enter the number of pieces of fresh fruit and dried fruit. one piece of fresh fruit, and two ‘pieces’ of dried fruit were counted as a serving. For vegetables, participants were asked to enter the number of heaped tablespoons of cooked vegetables and salad/raw vegetables eaten

per day. Two heaped tablespoons of vegetables were counted as a serving. We summed the servings of vegetable and fruit take, and >5 servings per day was considered the healthy category.

Table S1. Basic characteristics of colorectal cancer GWASs.

Stage	Population	GWAS study	Variables	Cases	Controls
Derivation	EAS/Chinese	NJCRC		1,316	2,207
			Sex		
			Male	794	1,290
			Female	522	917
			Age (year), mean ± SD	58.34 ± 12.85	57.87 ± 21.75
		BJCRC		932	966
			Sex		
			Male	547	490
			Female	385	476
			Age (year), mean ± SD	61.76 ± 13.75	60.98 ± 12.91
		SHCRC		1,116	1,054
			Sex		
			Male	675	725
			Female	441	316
			Age (year), mean ± SD	60.62 ± 11.68	59.65 ± 10.61
		ZJCRC		1,046	1,184
			Sex		
			Male	603	1,055
			Female	443	129
			Age (year), mean ± SD	62.25 ± 11.74	56.76 ± 12.48
	EAS/Japanese	BBJ		7,062	195,745
			Sex		
			Male	4,496	97,655
			Female	2,566	98,090
			Age (year), mean ± SD	67.00 ± 10.2	61.6 ± 13.9
	EUR	GECCO		21,608	20,278
			Sex		
			Male	11,236	9,584
			Female	10,372	10,694
			Age (year), mean ± SD	63.76 ± 10.62	60.85 ± 11.65
	EUR	PLCO		2,065	67,500
			Sex		
			Male	1,126	30,710
			Female	939	36,780
Validation	EAS/Chinese	JSCRC		727	1,452
			Sex		
			Male	439	894
			Female	288	558
			Age (year), mean ± SD	61.13 ± 12.49	61.16 ± 10.98
	EUR	CORSA		1,289	1,284
			Sex		

	Male	837	832
	Female	452	452
	Age (year), mean ± SD	66.21 ± 10.73	58.02 ± 13.08

Note: GWAS, genome-wide association studies; EAS, East Asian population; EUR, European population; BBJ, BioBank Japan Project; GECCO, Genetics and Epidemiology of Colorectal Cancer Consortium; PLCO, Prostate, Lung, Colorectal and Ovarian cancer screening trial; CORSA, Colorectal Cancer Study of Austria.

Table S2. Basic characteristics of the UK Biobank cohort.

Variables		All participants (N = 355,543)	Colorectal cancer (N = 2,621)	Non-colorectal cancer (N = 352,922)
Sex	Male	167,517	1,555	165,962
	Female	188,026	1,066	186,960
Age[a] (year), mean ± SD		56.79 ± 7.95	60.93 ± 6.34	56.76 ± 7.95
BMI (kg/m^3), mean ± SD		27.42 ± 4.75	27.94 ± 4.59	27.42 ± 4.75
Smoking status	Yes	160,163	1,412	158,751
	No	194,203	1,203	193,000
	Missing	1,177	6	1,171
Drinking status	Yes	344,417	2,539	341,878
	No	10,821	78	10,743
	Missing	305	4	301
Assessment center	Stockport (pilot)	338	4	334
	Manchester	9,492	92	9,400
	Oxford	10,253	95	10,158
	Cardiff	13,566	117	13,449
	Glasgow	13,463	115	13,348
	Edinburgh	13,044	111	12,933
	Stoke	14,997	116	14,881
	Reading	21,633	173	21,460
	Bury	21,313	175	21,138
	Newcastle	28,388	218	28,170
	Leeds	32,503	232	32,271
	Bristol	32,404	240	32,164
	Barts	5,968	43	5,925
	Nottingham	25,909	176	25,733
	Sheffield	23,250	144	23,106
	Liverpool	24,158	180	23,978
	Middlesborough	16,303	92	16,211
	Hounslow	14,908	101	14,807
	Croydon	15,172	89	15,083
	Birmingham	16,308	93	16,215
	Swansea	1,672	13	1,659
	Wrexham	501	2	499

[a] Age at baseline.
Note: BMI, body mass index.

Table S3. Summary of 37 colorectal cancer GWAS-reported SNPs in East Asian population.

SNP	Locus	Position[a]	Allele[b]	OR[c]	PMID[d]
rs201395236	1q44	245181421	T/C	1.75	30529582
rs7542665	1p31.3	62673037	C/T	1.08	30529582
rs7606562	2p16.3	48686695	T/A	1.10	30529582
rs113569514	3q22.2	133748789	T/C	1.10	30529582
rs12522693	5q23.3	130195731	A/G	1.31	26515597
rs12659017	5q23.2	125988175	G/A	1.09	30529582
rs647161	5q31.1	134499092	A/C	1.17	23263487
rs1476570	6p22.1	29809860	A/G	1.12	30529582
rs3830041	6p21.32	32191339	T/C	1.16	30529582
rs4711689	6p21.1	41692812	A/G	1.11	26965516
rs7758229*	6q25.3	160840252	T/G	1.28	21242260
rs2450115	8q23.3	117624093	T/C	1.12	26965516
rs6983267	8q24.21	128413305	G/T	1.18	21242260
rs10506868	10q25.2	114319380	T/C	1.10	26965516
rs11196172	10q25.2	114726843	A/G	1.14	24836286
rs1665650	10q25.3	118487100	T/C	1.13	23263487
rs4919687	10q24.32	104595248	G/A	1.14	26965516
rs704017	10q22.3	80819132	G/A	1.10	24836286
rs174537	11q12.2	61552680	G/T	1.16	24836286
rs10774214	12p13.32	4368352	T/C	1.17	23263487
rs10849432	12p13.31	6385727	T/C	1.14	24836286
rs11108175	12q22	96050887	A/G	1.08	31826910
rs2238126	12p13.2	12009741	G/A	1.17	27145994
rs2730985	12q12	43130624	G/A	1.08	30529582
rs77969132	12p11.21	31594813	T/C	1.44	30529582
rs9634162	12q24.21	115098094	A/G	1.07	31826910
rs1886450	13q22.1	73986628	G/A	1.09	30529582
rs4341754	16q23.2	80039621	G/C	1.09	30529582
rs847208	16q24.1	86254051	A/C	1.11	29471430
rs12603526	17p13.3	800593	C/T	1.10	24836286
rs17836917	17q12	32047282	G/A	1.33	26515597
rs7229639	18q21.1	46450976	A/G	1.22	24448986
rs2241714	19q13.2	41869392	C/T	1.09	24836286
rs13831	20q13.32	57475191	G/A	1.08	30529582
rs2423279	20p12.3	7812350	C/T	1.14	23263487
rs6061231	20q13.33	60956917	C/A	1.18	26965516
rs6065668	20q13.12	42532821	T/C	1.11	29471430

[a] Chromosomal position, hg19/GRCh37 build.

[b] Risk/reference allele.

[c] Reported OR value from previous East Asian GWASs.

[d] PubMed ID.

*Associated with distal colon cancer risk in GWAS.

Note: GWAS, genome-wide association study; SNP, single nucleotide polymorphism; OR, odds ratio; 95% CI, 95% confidence interval; PRS, polygenic risk score.

Table S4. Summary of eight lifestyle factors in the UK Biobank cohort.

Lifestyle factor[a]	All participants (N = 355,543)	Colorectal cancer (N = 2,621)	Non-colorectal cancer (N = 352,922)	HR (95% CI)[b]	*P*[b]
BMI					
1	111,276	672	110,604	0.88 (0.80, 0.96)	0.004
0	243,149	1,938	241,211	1.00 (reference)	
Missing	1,118	11	1,107		
Smoking status					
1	194,203	1,203	193,000	0.82 (0.76, 0.88)	3.58E-07
0	160,163	1,412	158,751	1.00 (reference)	
Missing	1,177	6	1,171		
Drinking status					
1	10,821	78	10,743	0.93 (0.74, 1.17)	0.551
0	344,417	2,539	341,878	1.00 (reference)	
Missing	305	4	301		
WHR					
1	178,482	961	177,521	0.75 (0.69, 0.82)	1.60E-10
0	176,418	1,654	174,764	1.00 (reference)	
Missing	643	6	637		
Physical activity					
1	47,581	325	47,256	0.90 (0.80, 1.01)	0.065
0	281,439	2,052	279,387	1.00 (reference)	
Missing	26,523	244	26,279		
Sedentary time					
1	100,797	598	100,199	0.91 (0.83, 1.00)	0.050
0	250,048	1,982	248,066	1.00 (reference)	
Missing	4,698	41	4,657		
Red and processed meat intake					
1	204,545	1,335	203,210	0.84 (0.77, 0.90)	7.75E-06
0	147,507	1,262	146,245	1.00 (reference)	
Missing	3,491	24	3,467		
Vegetable and fruit intake					
1	135,142	995	134,147	0.96 (0.89, 1.04)	0.369
0	211,808	1,549	210,259	1.00 (reference)	
Missing	8,593	77	8,516		

[a] Each lifestyle factor was given a score of 0 or 1, with 1 representing the healthy category (BMI, 18.5-24.9 kg/m^3; smoking status, never; drinking status, never; WHR, < 0.85 for women, or < 0.90 for men; physical activity, 6–7 days per week of moderate activity and 3–5 days per week of vigorous activity, or 6–7 days per week of vigorous activity; sedentary time, < 3 hours per day; red and processed meat intake, < 4 times per week; vegetable and fruit intake, > 5 servings per day). Note: BMI, body mass index. WHR, waist-to-hip ratio.

[b] With the adjustment of sex, age, center and first 10 principal components.

Note: BMI, body mass index; WHR, waist-to-hip ratio.

Table S5. Summary of one novel EAS-EUR conditionally independent variant at known colorectal cancer risk loci.

Locus	SNP	Position[a]	Allele[b]	RAF[c]		Ancestry[d]	OR (95% CI)	*P*	I^2	P_{het}[e]	$OR_{conditional}$ (95% CI)[f]	*P*[f]
				EAS	EUR							
3p14.1	rs7623129	64624426	C/T	0.455	0.534	EAS	1.05 (1.02, 1.09)	0.002				
						EUR	1.06 (1.04, 1.09)	3.66E-06			1.06 (1.04, 1.08)	1.18E-08
						Combined	1.06 (1.04, 1.08)	2.68E-08	0%	0.714		

[a] Chromosomal position, hg19/GRCh37 build.

[b] Risk/reference allele.

[c] Risk allele frequency from the 1000 Genomes Project (Phase 3) used in this study.

[d] Combined: Meta-analysis.

[e] *P* value for heterogeneity test.

[f] Conditional analysis on previously GWAS variants on 3p14.1.

Note: EAS, East Asian; EUR, European; OR, odds ratio; 95% CI, 95% confidence interval; GWAS, genome-wide association study; SNP, single nucleotide polymorphism.

Table S6. Functional annotations of one novel colorectal cancer risk locus.

Locus	SNP	Nearest gene	Enhancer histone marks	DNAse	Regulome DB score[a]	CADD (Phred)	Nearby egenes[c]
3p14.1	rs7623129	*ADAMTS9*	ESDR, ESC, BRN	BRN,BRN	5	< 5	*ADAMTS9*

[a] Regulome DB Score: 5, TF binding or DNase peak.

[c] egenes were identified using eQTL analysis with P value < 0.05 in sigmoid or transverse tissues.

Table S7. The association of PRS with colorectal cancer risk in the UK Biobank cohort.

Model	PRS	Cases/All	HR (95% CI)	*P*	P_{trend}
Model 1[a]	Continuous	Per SD	1.42 (1.37, 1.48)	3.53E-72	
	Low	121/35,555	1.00 (reference)		
	Intermediate	2,036/284,433	2.11 (1.76, 2.54)	1.30E-15	
	High	464/35,555	3.88 (3.18, 4.74)	2.82E-40	8.15E-53
Model 2[b]	Continuous	Per SD	1.42 (1.36, 1.48)	1.72E-63	
	Low	121/35,555	1.00 (reference)		
	Intermediate	2,036/284,433	2.22 (1.82, 2.71)	5.32E-15	
	High	464/35,555	3.92 (3.16, 4.88)	9.46E-35	3.58E-44

[a] Model 1, based on Cox regression model with the adjustment of sex, age, center and first 10 principal components.

[b] Model 2, based on Cox regression model with the adjustment of sex, age, center, lifestyle score and first 10 principal components.

Note: PRS, polygenic risk score; HR, hazard ratio; 95% CI, 95% confidence interval; SD, standard deviation.

Table S8. Sensitivity analyses for the association of PRS with colorectal cancer risk in the UK Biobank cohort.

Sensitivity analyses	Model	PRS	Cases/All	HR (95% CI)	P	P_{trend}
Excluded colorectal cancer patients occurred within the first year of follow-up	Model 1[a]	Low	108/35,542	1.00 (reference)		
		Intermediate	1,812/284,209	2.11 (1.73, 2.56)	5.41E-14	
		High	415/35,506	3.89 (3.15, 4.81)	2.86E-36	1.04E-47
	Model 2[b]	Low	108/35,542	1.00 (reference)		
		Intermediate	1,812/284,209	2.21 (1.79, 2.72)	2.15E-13	
		High	415/35,506	3.98 (3.16, 5.01)	7.72E-32	5.07E-41
Ancestry-corrected PRS	Model 1[c]	Low	121/35,555	1.00 (reference)		
		Intermediate	2,041/284,433	2.13 (1.77, 2.55)	7.88E-16	
		High	459/35,555	3.87 (3.16, 4.72)	5.78E-40	7.06E-52
	Model 2[d]	Low	121/35,555	1.00 (reference)		
		Intermediate	2,041/284,433	2.20 (1.81, 2.69)	6.24E-15	
		High	459/35,555	3.87 (3.12, 4.81)	2.51E-34	2.72E-43
Excluded non-colorectal cancer individuals with other cancers occurred within the time of follow-up	Model 1[a]	Low	121/33,315	1.00 (reference)		
		Intermediate	2,036/265,424	2.12 (1.77, 2.55)	8.21E-16	
		High	464/33,114	3.91 (3.2, 4.78)	9.87E-41	2.15E-53
	Model 2[b]	Low	121/33,315	1.00 (reference)		
		Intermediate	2,036/265,424	2.23 (1.83, 2.72)	3.46E-15	
		High	464/33,114	3.96 (3.18, 4.92)	3.46E-35	9.57E-45

[a] Model 1, based on Cox regression model with the adjustment of sex, age, center and first 10 principal components.

[b] Model 2, based on Cox regression model with the adjustment of sex, age, center, lifestyle score and first 10 principal components.

[c] Model 1, based on Cox regression model with the adjustment of sex, age and center.

[d] Model 2, based on Cox regression model with the adjustment of sex, age, center and lifestyle score.

Note: PRS, polygenic risk score; HR, hazard ratio; 95% CI, 95% confidence interval.

Table S9. The association of lifestyle score with colorectal cancer risk in the UK Biobank cohort.

Model	Lifestyle	Cases/All	HR (95% CI)	P	P_{trend}
Model 1[a]	Continuous	Per score	0.90 (0.88, 0.93)	3.39E-12	
	Unfavorable	713/68,426	1.00 (reference)		
	Intermediate	1,035/144,059	0.79 (0.72, 0.87)	2.86E-06	
	Favorable	544/105,456	0.65 (0.58, 0.74)	2.56E-12	1.92E-12
Model 2[b]	Continuous	Per score	0.90 (0.88, 0.93)	9.69E-12	
	Unfavorable	713/68,426	1.00 (reference)		
	Intermediate	1,035/144,059	0.79 (0.72, 0.88)	3.65E-06	
	Favorable	544/105,456	0.66 (0.58, 0.74)	7.17E-12	5.34E-12

[a] Model 1, based on Cox regression model with the adjustment of sex, age, center and first 10 principal components.

[b] Model 2, based on Cox regression model with the adjustment of sex, age, center, polygenic risk score (PRS) and first 10 principal components.

Note: HR, hazard ratio; 95% CI, 95% confidence interval.

Table S10. Sensitivity analyses for the association of lifestyle score with colorectal cancer risk in the UK Biobank cohort.

Sensitivity analyses	Model	Lifestyle	Cases/All	HR (95% CI)	P	P_{trend}
Excluded colorectal cancer patients occurred within the first year of follow-up	Model 1[a]	Unfavorable	631/68,344	1.00 (reference)		
		Intermediate	920/143,944	0.79 (0.71, 0.88)	8.45E-06	
		Favorable	490/105,402	0.66 (0.58, 0.74)	5.56E-11	4.40E-11
	Model 2[b]	Unfavorable	631/68,344	1.00 (reference)		
		Intermediate	920/143,944	0.79 (0.71, 0.88)	1.07E-05	
		Favorable	490/105,402	0.66 (0.58, 0.75)	1.41E-10	1.11E-10
Reclassified lifestyle categories	Model 1[a]	Unfavorable	1,274/139,616	1.00 (reference)		
		Intermediate	780/130,317	0.78 (0.71, 0.85)	9.97E-08	
		Favorable	238/48,008	0.71 (0.61, 0.82)	2.94E-06	2.43E-09
	Model 2[b]	Unfavorable	1,274/139,616	1.00 (reference)		
		Intermediate	780/130,317	0.78 (0.71, 0.86)	1.36E-07	
		Favorable	238/48,008	0.72 (0.62, 0.83)	5.37E-06	5.06E-09
Excluded non-colorectal cancer individuals with other cancers occurred within the time of follow-up	Model 1[a]	Unfavorable	713/63,031	1.00 (reference)		
		Intermediate	1,035/134,596	0.79 (0.71, 0.87)	1.31E-06	
		Favorable	544/99,542	0.64 (0.57, 0.73)	4.43E-13	3.28E-13
	Model 2[b]	Unfavorable	713/63,031	1.00 (reference)		
		Intermediate	1,035/134,596	0.79 (0.71, 0.87)	1.77E-06	
		Favorable	544/99,542	0.65 (0.58, 0.73)	1.37E-12	1.01E-12

[a] Model 1, based on Cox regression model with the adjustment of sex, age, center and first 10 principal components.

[b] Model 2, based on Cox regression model with the adjustment of sex, age, center, polygenic risk score (PRS) and first 10 principal components.

Note: HR, hazard ratio; 95% CI, 95% confidence interval.

Table S11. Sensitivity analyses for cumulative risk of developing colorectal cancer according to different levels of PRS and lifestyle score in the UK Biobank cohort.

Sensitivity analyses	PRS	Lifestyle	Cases/All	Incidence proportion (95% CI)	HR (95% CI)[a]	P^{a}	P_{trend}
Excluded colorectal cancer patients occurred within the first year of follow-up	Low	Unfavorable	26/6,643	0.39% (0.26, 0.57)	1.00 (reference)		
		Intermediate	44/14,335	0.31% (0.22, 0.41)	0.89 (0.54, 1.46)	0.641	
		Favorable	21/10,912	0.19% (0.12, 0.29)	0.64 (0.35, 1.19)	0.157	0.157
	Intermediate	Unfavorable	499/54,678	0.91% (0.83, 1.00)	1.00 (reference)		
		Intermediate	720/115,227	0.62% (0.58, 0.67)	0.78 (0.7, 0.88)	4.77E-05	
		Favorable	376/84,288	0.45% (0.4, 0.49)	0.64 (0.56, 0.74)	1.12E-09	8.89E-10
	High	Unfavorable	106/7,023	1.51% (1.24, 1.83)	1.00 (reference)		
		Intermediate	156/14,382	1.08% (0.92, 1.27)	0.80 (0.62, 1.03)	0.083	
		Favorable	93/10,202	0.91% (0.74, 1.12)	0.76 (0.56, 1.02)	0.066	0.066
Ancestry-corrected PRS	Low	Unfavorable	34/6,668	0.51% (0.35, 0.71)	1.00 (reference)		
		Intermediate	46/14,326	0.32% (0.24, 0.43)	0.73 (0.46, 1.15)	0.176	
		Favorable	23/10,903	0.21% (0.13, 0.32)	0.57 (0.32, 1)	0.049	0.047
	Intermediate	Unfavorable	565/54,752	1.03% (0.95, 1.12)	1.00 (reference)		
		Intermediate	810/115,269	0.70% (0.66, 0.75)	0.78 (0.7, 0.87)	1.38E-05	
		Favorable	424/84,360	0.50% (0.46, 0.55)	0.64 (0.56, 0.74)	1.57E-10	1.18E-10
	High	Unfavorable	114/7,006	1.63% (1.34, 1.95)	1.00 (reference)		
		Intermediate	179/14,464	1.24% (1.06, 1.43)	0.86 (0.67, 1.09)	0.206	
		Favorable	97/10,193	0.95% (0.77, 1.16)	0.75 (0.56, 1)	0.052	0.052
Reclassified lifestyle categories	Low	Unfavorable	64/13,827	0.46% (0.36, 0.59)	1.00 (reference)		
		Intermediate	31/12,984	0.24% (0.16, 0.34)	0.60 (0.39, 0.94)	0.025	
		Favorable	7/5,090	0.14% (0.06, 0.28)	0.39 (0.17, 0.86)	0.020	0.004

	Intermediate	Unfavorable	1,006/111,636	0.90% (0.85, 0.96)	1.00 (reference)		
		Intermediate	606/10,4451	0.58% (0.53, 0.63)	0.77 (0.69, 0.85)	6.03E-07	
		Favorable	185/38,308	0.48% (0.42, 0.56)	0.70 (0.6, 0.83)	2.39E-05	4.27E-08
	High	Unfavorable	204/14,153	1.44% (1.25, 1.65)	1.00 (reference)		
		Intermediate	143/12,882	1.11% (0.94, 1.31)	0.90 (0.72, 1.12)	0.339	
		Favorable	46/4,610	1.00% (0.73, 1.33)	0.88 (0.63, 1.23)	0.458	0.328
Excluded non-colorectal cancer individuals with other cancers occurred within the time of follow-up	Low	Unfavorable	33/6,156	0.54% (0.37, 0.75)	1.00 (reference)		
		Intermediate	47/13,433	0.35% (0.26, 0.47)	0.76 (0.48, 1.2)	0.235	
		Favorable	22/10,350	0.21% (0.13, 0.32)	0.54 (0.31, 0.97)	0.038	0.037
	Intermediate	Unfavorable	567/50,412	1.12% (1.03, 1.22)	1.00 (reference)		
		Intermediate	808/107,751	0.75% (0.70, 0.80)	0.77 (0.69, 0.86)	4.61E-06	
		Favorable	422/79,576	0.53% (0.48, 0.58)	0.63 (0.55, 0.72)	2.41E-11	1.72E-11
	High	Unfavorable	113/6,463	1.75% (1.44, 2.10)	1.00 (reference)		
		Intermediate	180/13,412	1.34% (1.15, 1.55)	0.86 (0.68, 1.1)	0.232	
		Favorable	100/9,616	1.04% (0.85, 1.26)	0.76 (0.57, 1.01)	0.061	0.061

[a] Derived from Cox regression model with the adjustment of sex, age, center and first 10 principal components.

Note: PRS, polygenic risk score; HR, hazard ratio; 95% CI, 95% confidence interval.

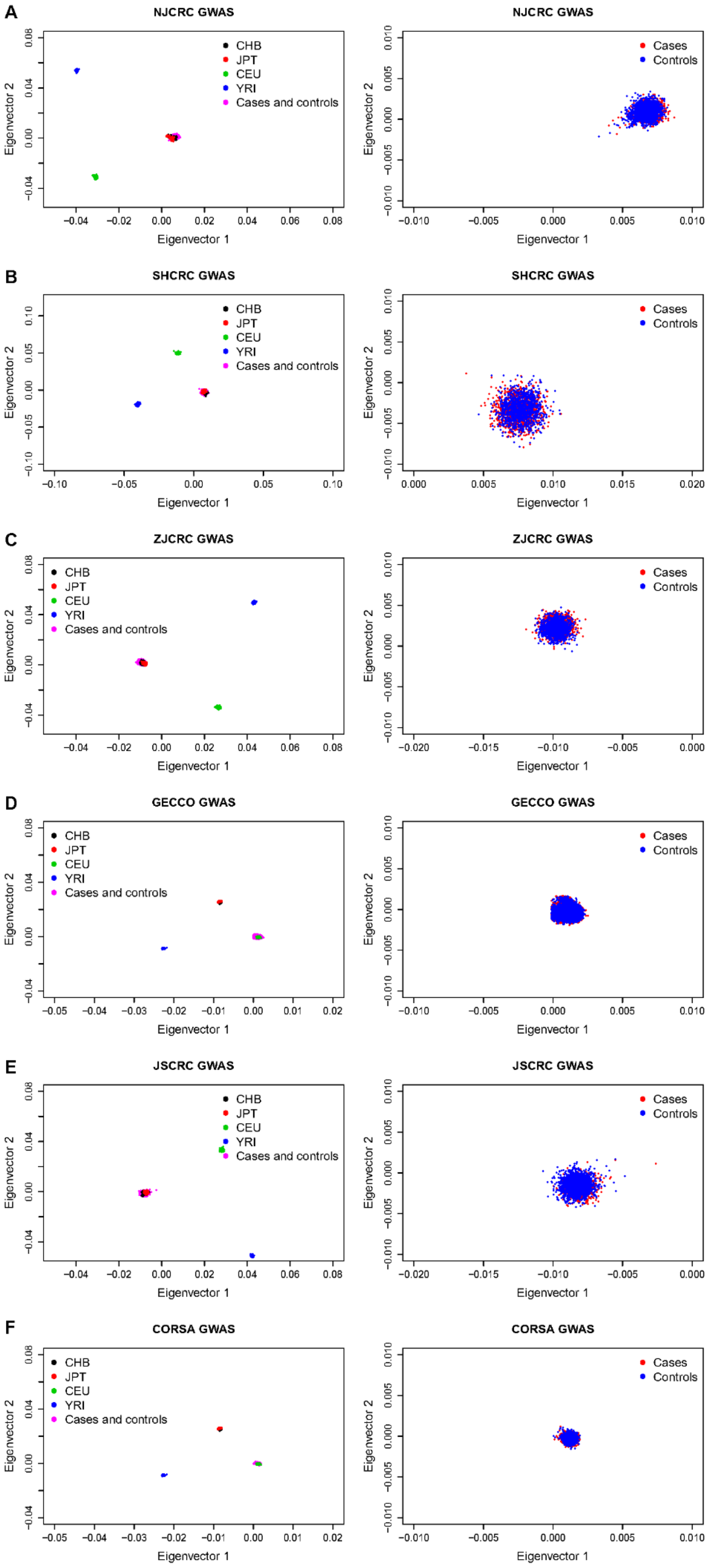
A
NJCRC GWAS
CHB
JPT
CEU
YRI
Cases and controls
Cases
Controls
Eigenvector 1
Eigenvector 2
B
SHCRC GWAS
C
ZJCRC GWAS
D
GECCO GWAS
E
JSCRC GWAS
F
CORSA GWAS

Fig. S1. Principal component analysis based on the colorectal cancer GWAS subjects and 1000 Genomes Project populations. (A) NJCRC GWAS of derivation stage; (B) SHCRC GWAS of derivation stage; (C) ZJCRC GWAS of derivation stage; (D) GECCO GWAS of derivation stage; (E) JSCRC GWAS of validation stage and (F) CORSA GWAS of validation stage.

Note: CHB, Han Chinese in Beijing, China; JPT, Japanese in Tokyo, Japan; CEU, Utah residents with Northern and Western European ancestry; YRI, Yoruba in Ibadan, Nigeria; GWAS, genome-wide association study; GECCO, Genetics and Epidemiology of Colorectal Cancer Consortium; CORSA, Colorectal Cancer Study of Austria.

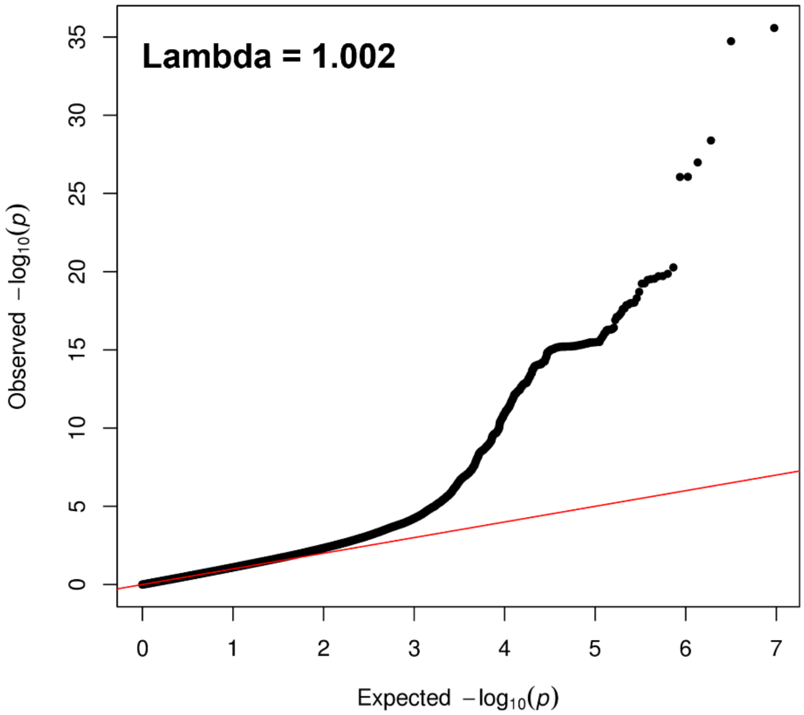

Fig. S2. Quantile-quantile plot and genomic inflation factor for the association with colorectal cancer risk in the meta-analysis of EAS-EUR GWASs. The X axis shows the expected distribution of the observed -$\log_{10}$ (*P* values) under the null hypothesis of no association. The Y axis shows the distribution of the observed -$\log_{10}$ (*P* values) of meta-analysis. Genomic inflation values (i.e., lambda) were adjusted as equal to a study of 1,000 cases and 1,000 controls.

Note: GWAS, genome-wide association study.

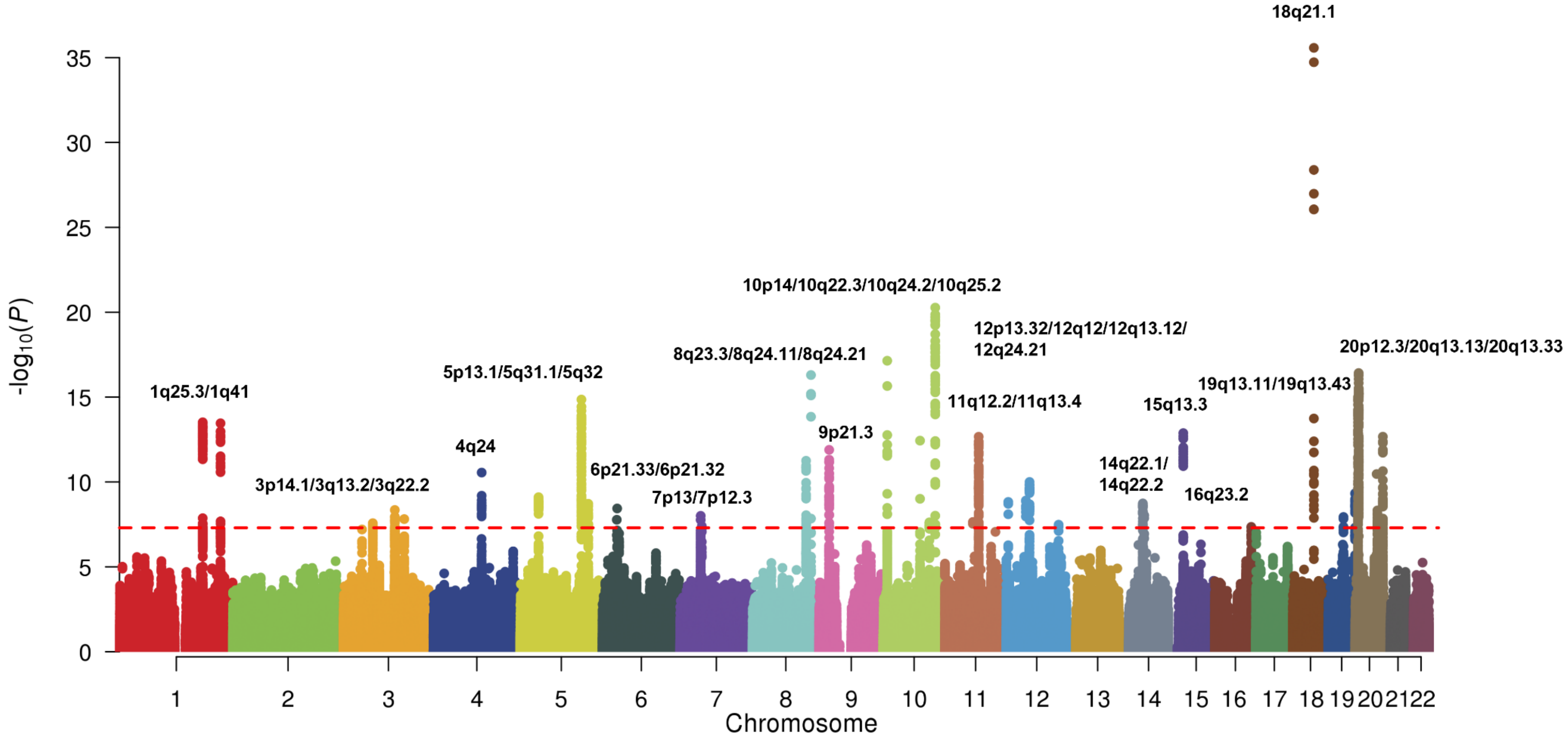

Fig. S3. Manhattan plot from colorectal cancer EAS-EUR GWAS meta-analysis. The associations (-$\log_{10}$ (*P*-value) values, Y-axis) are plotted against genomic position (X-axis by chromosome and the chromosomal position of NCBI build 37). The red dashed line indicates the genome-wide significance threshold ($P = 5\times10^{-8}$). Note: GWAS, genome-wide association study.

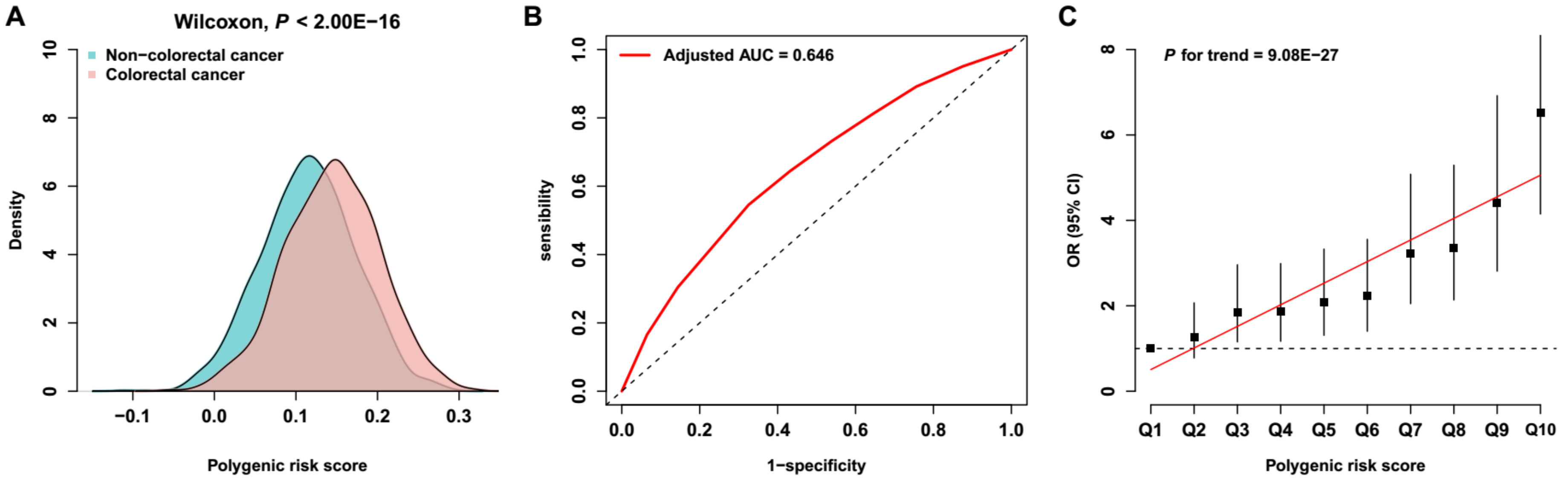

Fig. S4. The association of PRS_{CSx} with incident colorectal cancer in the JSCRC GWAS dataset. (A) Density curve of PRS among colorectal cancer and non-colorectal cancer individuals. The *P* value was calculated by Wilcoxon rank-sum test. (B) Covariates-adjusted receiver operating characteristics curve for PRS model. (C) Participants were divided into ten equal groups according to the distribution of PRS, and the OR and 95% CI of each group were calculated compared with those at the lowest tenth group with the adjustment of sex, age and principal components.

Note: PRS, polygenic risk score; OR, odds ratio; 95% CI, 95% confidence interval.

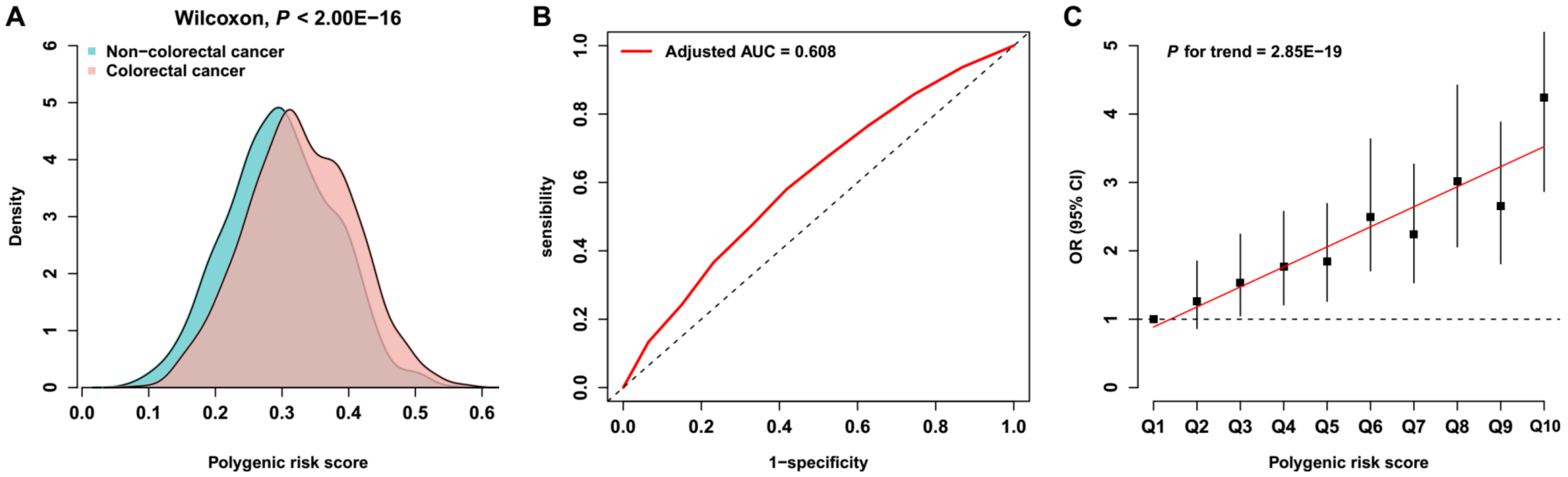

Fig. S5. The association of PRS_{CSx} with incident colorectal cancer in the CORSA GWAS dataset. (A) Density curve of PRS among colorectal cancer and non-colorectal cancer individuals. The *P* value was calculated by Wilcoxon rank-sum test. (B) Covariates-adjusted Receiver operating characteristics curve for PRS model. (C) Participants were divided into ten equal groups according to the distribution of PRS, and the OR and 95% CI of each group were calculated compared with those at the lowest tenth group with the adjustment of sex, age and principal components.

Note: PRS, polygenic risk score; OR, odds ratio; 95% CI, 95% confidence interval; CORSA, Colorectal Cancer Study of Austria.

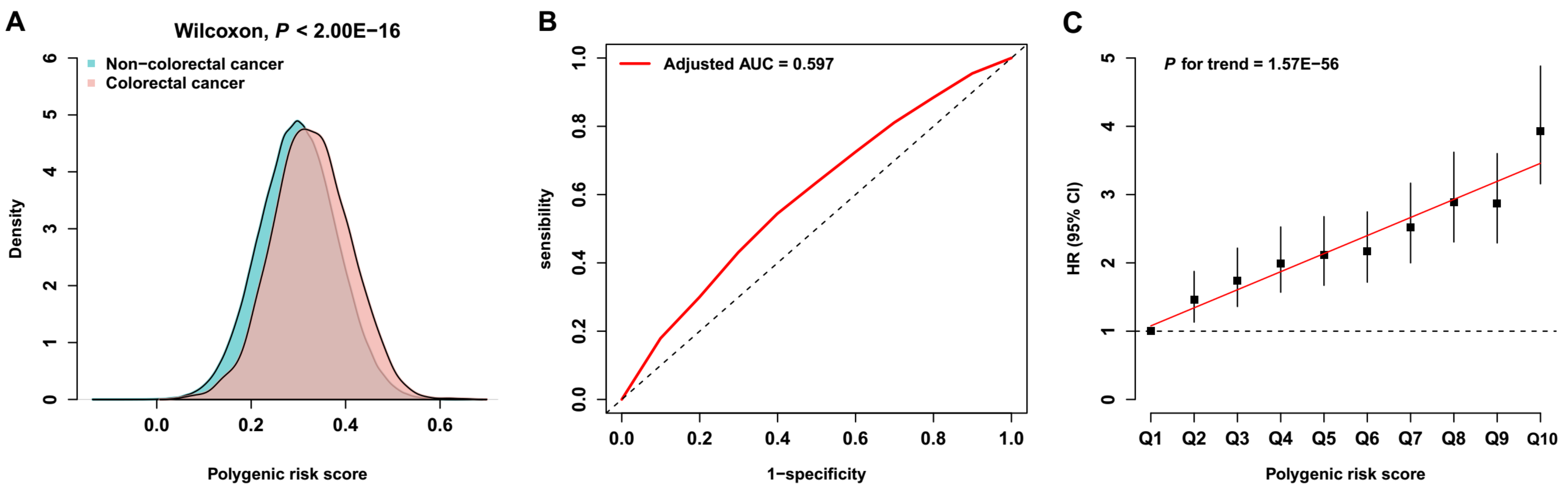

Fig. S6. The association of PRS with incident colorectal cancer in the UK Biobank cohort. (A) Density curve of PRS among colorectal cancer and non-colorectal cancer individuals. The *P* value was calculated by Wilcoxon rank-sum test. (B) Covariates-adjusted receiver operating characteristics curve for PRS model. (C) Participants were divided into ten equal groups according to the distribution of PRS, and the HR and 95% CI of each group were calculated compared with those at the lowest tenth group with the adjustment of sex, age, center, lifestyle score and first 10 principal components.

Note: PRS, polygenic risk score; HR, hazard ratio; 95% CI, 95% confidence interval.

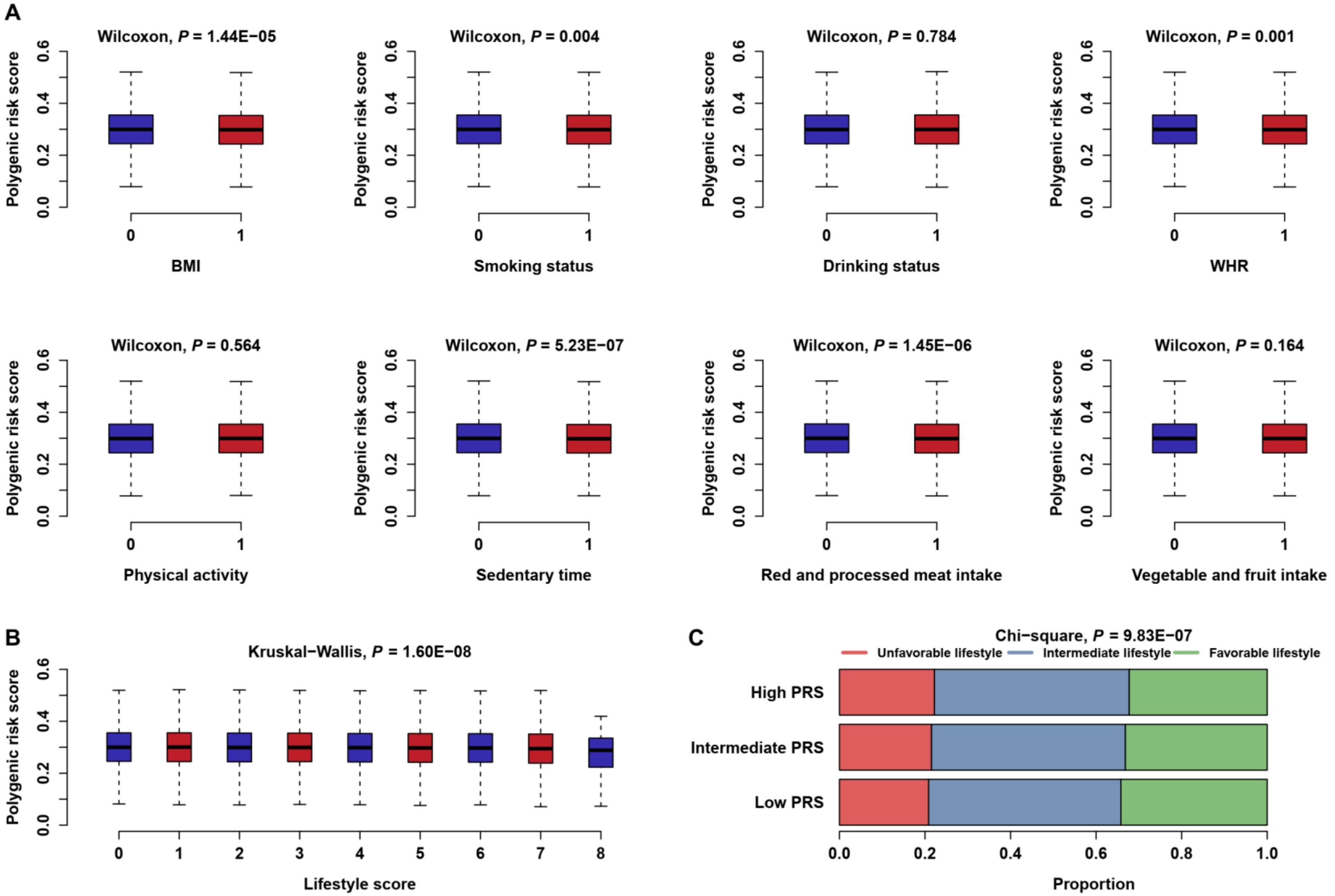
A
Wilcoxon, P = 1.44E−05
Wilcoxon, P = 0.004
Wilcoxon, P = 0.784
Wilcoxon, P = 0.001
Polygenic risk score
BMI
Smoking status
Drinking status
WHR
Wilcoxon, P = 0.564
Wilcoxon, P = 5.23E−07
Wilcoxon, P = 1.45E−06
Wilcoxon, P = 0.164
Physical activity
Sedentary time
Red and processed meat intake
Vegetable and fruit intake
B
Kruskal−Wallis, P = 1.60E−08
Lifestyle score
C
Chi−square, P = 9.83E−07
Unfavorable lifestyle
Intermediate lifestyle
Favorable lifestyle
High PRS
Intermediate PRS
Low PRS
Proportion

Fig. S7. The association of PRS with lifestyle factors in the UK Biobank cohort. (A) Each lifestyle factor (given a score of 0 or 1, with 1 representing the healthy category) and PRS; (B) Lifestyle score and PRS; (C) Different levels of lifestyle score and PRS. The *P* value was calculated by Wilcoxon rank-sum test, Kruskal-Wallis test or Chi-square test.

Note: PRS, polygenic risk score; BMI, body mass index; WHR, waist-to-hip ratio.

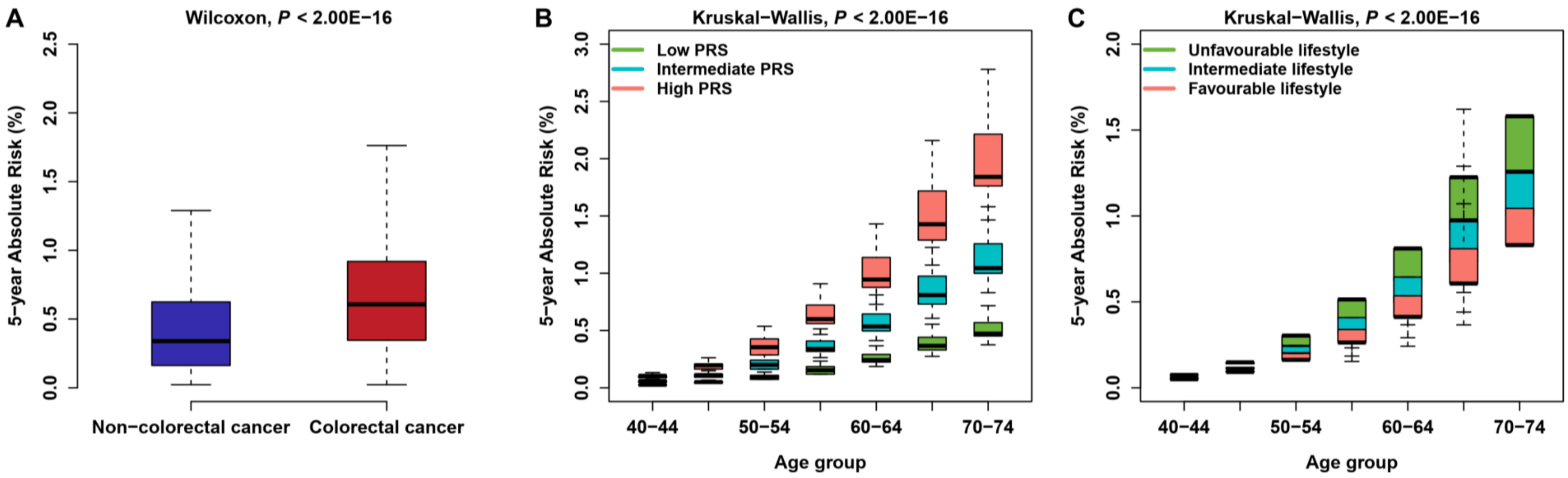

Fig. S8. Distribution of 5-year absolute risk of developing colorectal cancer in the UK Biobank cohort. (A) Boxplot of 5-year absolute risk among colorectal cancer and non-colorectal cancer individuals. The *P* value was calculated by Wilcoxon rank-sum test. (B-C) Boxplots of 5-year absolute risk stratified by different levels of (B) PRS and (C) lifestyle score among different age (i.e., age of cohort entry) groups. The *P* value was calculated by Kruskal-Wallis test.

Note: PRS, polygenic risk score.

References:

1. Xin J, Du M, Gu D, Ge Y, Li S, Chu H, et al. Combinations of single nucleotide polymorphisms identified in genome-wide association studies determine risk for colorectal cancer. Int J Cancer. 2019; 145(10):2661-2669.
2. Xin J, Chu H, Ben S, Ge Y, Shao W, Zhao Y, et al. Evaluating the effect of multiple genetic risk score models on colorectal cancer risk prediction. Gene. 2018; 673:174-180.
3. Jiang K, Sun Y, Wang C, Ji J, Li Y, Ye Y, et al. Genome-wide association study identifies two new susceptibility loci for colorectal cancer at 5q23.3 and 17q12 in Han Chinese. Oncotarget. 2015; 6(37):40327-40336.
4. Qu X, Zhao L, Wang M, Zhang R, Cheng L, Qiu L, et al. Novel functional variants in the Notch pathway and survival of Chinese colorectal cancer. Int J Cancer. 2021; 149(1):84-96.
5. Ishigaki K, Akiyama M, Kanai M, Takahashi A, Kawakami E, Sugishita H, et al. Large-scale genome-wide association study in a Japanese population identifies novel susceptibility loci across different diseases. Nat Genet. 2020; 52(7):669-679.
6. Huyghe JR, Bien SA, Harrison TA, Kang HM, Chen S, Schmit SL, et al. Discovery of common and rare genetic risk variants for colorectal cancer. Nat Genet. 2019; 51(1):76-87.
7. Peters U, Jiao S, Schumacher FR, Hutter CM, Aragaki AK, Baron JA, et al. Identification of Genetic Susceptibility Loci for Colorectal Tumors in a Genome-Wide Meta-analysis. Gastroenterology. 2013; 144(4):799-807.
8. Chu H, Xin J, Yuan Q, Wu Y, Du M, Zheng R, et al. A prospective study of the associations among fine particulate matter, genetic variants, and the risk of colorectal cancer. Environ Int. 2021; 147:106309.
9. Bycroft C, Freeman C, Petkova D, Band G, Elliott LT, Sharp K, et al. The UK Biobank resource with deep phenotyping and genomic data. Nature. 2018; 562(7726):203-209.
10. Sudlow C, Gallacher J, Allen N, Beral V, Burton P, Danesh J, et al. UK biobank:

an open access resource for identifying the causes of a wide range of complex diseases of middle and old age. Plos Med. 2015; 12(3):e1001779.

11. Thomas M, Sakoda LC, Hoffmeister M, Rosenthal EA, Lee JK, van Duijnhoven F, et al. Genome-wide Modeling of Polygenic Risk Score in Colorectal Cancer Risk. Am J Hum Genet. 2020; 107(3):432-444.
12. Choi SW, Mak TS, O'Reilly PF. Tutorial: a guide to performing polygenic risk score analyses. Nat Protoc. 2020; 15(9):2759-2772.
13. Vilhjalmsson BJ, Yang J, Finucane HK, Gusev A, Lindstrom S, Ripke S, et al. Modeling Linkage Disequilibrium Increases Accuracy of Polygenic Risk Scores. Am J Hum Genet. 2015; 97(4):576-592.
14. Mak T, Porsch RM, Choi SW, Zhou X, Sham PC. Polygenic scores via penalized regression on summary statistics. Genet Epidemiol. 2017; 41(6):469-480.
15. Prive F, Arbel J, Vilhjalmsson BJ. LDpred2: better, faster, stronger. Bioinformatics. 2020; 36(22-23):5424-5431.
16. Ruan Y, Lin YF, Feng YA, Chen CY, Lam M, Guo Z, et al. Improving polygenic prediction in ancestrally diverse populations. Nat Genet. 2022; 54(5):573-580.
17. Ge T, Irvin MR, Patki A, Srinivasasainagendra V, Lin YF, Tiwari HK, et al. Development and validation of a trans-ancestry polygenic risk score for type 2 diabetes in diverse populations. Genome Med. 2022; 14(1):70.
